# Exosome Carrier Effects; Resistance to Digestion in Phagolysosomes May Assist Transfers to Targeted Cells; II Transfers of miRNAs Are Better Analyzed via Systems Approach as They Do Not Fit Conventional Reductionist Stoichiometric Concepts

**DOI:** 10.3390/ijms23116192

**Published:** 2022-05-31

**Authors:** Philip W. Askenase

**Affiliations:** Section of Rheumatology, Allergy and Clinical Immunology, Department of Internal Medicine, School of Medicine, Yale University, 217 TAC Building South, 333 Cedar Street, New Haven, CT 06520, USA; philip.askenase@yale.edu; Tel.: +1-203-809-2013

**Keywords:** exosomes, micro vesicles, extracellular vesicles, miRNA, systems biology, antigen affinity columns

## Abstract

Carrier effects of extracellular vesicles (EV) like exosomes refer to properties of the vesicles that contribute to the transferred biologic effects of their contents to targeted cells. This can pertain to ingested small amounts of xenogeneic plant miRNAs and oral administration of immunosuppressive exosomes. The exosomes contribute carrier effects on transfers of miRNAs by contributing both to the delivery and the subsequent functional intracellular outcomes. This is in contrast to current quantitative canonical rules that dictate just the minimum copies of a miRNA for functional effects, and thus successful transfers, independent of the EV carrier effects. Thus, we argue here that transfers by non-canonical minute quantities of miRNAs must consider the EV carrier effects of functional low levels of exosome transferred miRNA that may not fit conventional reductionist stoichiometric concepts. Accordingly, we have examined traditional stoichiometry vs. systems biology that may be more appropriate for delivered exosome functional responses. Exosome carrier properties discussed include; their required surface activating interactions with targeted cells, potential alternate targets beyond mRNAs, like reaching a threshold, three dimensional aspects of the RNAs, added EV kinetic dynamic aspects making transfers four dimensional, and unique intracellular release from EV that resist intracellular digestion in phagolysosomes. Together these EV carrier considerations might allow systems analysis. This can then result in a more appropriate understanding of transferred exosome carrier-assisted functional transfers. A plea is made that the miRNA expert community, in collaboration with exosome experts, perform new experiments on molecular and quantitative miRNA functional effects in systems that include EVs, like variation in EV type and surface constituents, delivery, dose and time to hopefully create more appropriate and truly current canonical concepts of the consequent miRNA functional transfers by EVs like exosomes.

## 1. Introduction

The prior paper on this subject proposed that intercellular functional transfers of miRNAs via extracellular vesicles (EV), like exosomes that seem impossible according to current concepts, are dependent on carrier effects of the exosomes themselves that were not considered previously.

Carrier effects identified included the great variety of various EV subtypes, and the survival of exosomes under very harsh conditions like hypoxia and in the highly acidic and digestive enzyme-rich environment of the stomach. Further reviewed were quantitative carrier effects, like the minute attoliter volume of exosomes that cause a high concentration of the contained low-doses of miRNAs.

Additional quantitative carrier effects were that only a small subset of the applied exosomes actually participated in the miRNA transfers, and furthermore, only a small subset of the total recipient cell population was actually targeted for the miRNA transfers. Together, these carrier properties of exosomes were proposed to enable low dose miRNA transfers, not contemplated as possible by classical canonical thinking that does not consider the biologic carrier roles of the transferring EV.

In this follow up paper, numerous exosome surface activations of target cells are considered as carrier effects, as are intracellular properties of the delivering exosomes; like their intracellular resistance to the acid/enzyme rich environment of phagolysosomes, that are postulated to confer augmented actions on contained low-dose miRNAs. Furthermore, we suggest that functional low-level transfer of miRNAs do not fit conventional reductionist stoichiometric concepts. As an alternate, it is proposed that Systems Biology Concepts are more appropriate to the multiple biologic carrier, dependent actions that can result in epigenetic actions of the exosome transferred miRNAs.

## 2. Regarding Nomenclature: EVs vs. Exosomes

Although, there is a published opinion that at the moment the term extracellular vesicle (EV) is recommended for the natural vesicles discussed here [1], the term exosomes is preferred as the generic term for the main functional subset of released nanovesicles that transfer miRNAs.

The alternate recommended term, “Small EV” is not a solution as “small” is an indefinite word to be used strictly when comparing exosome to larger micro vesicles (MV). The functional biology of miRNA transfers, which is the focus here, is hardly ever ascribed to MV or even to smaller exomeres. The significant overlap anatomically of exosomes and apoptosis-derived amphisome vesicles, with both arising during intracellular formation via the endosomal pathway, means that the latter might be involved [2]. However, there are few or any recognized instances of productive transfer of RNAs by amphisomes, and they are about cell death, not life.

## 3. Low-Dose Delivered Exosome miRNAs Do Not Act like Hormones or Cytokines

Disagreements concerning exosome miRNA transfers have resulted in bewilderment and non-belief by conventional miRNA workers. This is a good thing. New data beyond the existing rules obtained from experiments on entirely new exosome carrier transfers of miRNA can lead to new non-canonical concepts. Disagreements can occur if one regards miRNA delivery to cells as acting like hormones or cytokines. Conventionally, these are unlike exosome transfers of genetic information via miRNAs. Instead, these agonists bind to specific cell surface receptors, triggering the stimulation of linked intracellular signaling cascades that mediate and regulate the consequent effects of the hormones or cytokines on gene expression involved [2].

In contrast to hormones and cytokines [3,4], the miRNAs transferred by carrier-acting exosomes are not distributed homogenously in the blood, but confined in the small volume of EV, such as exosomes. Surface delivery of these natural nano particles to targeted cells, followed by the classical binding of their carried miRNA to cytoplasmic mRNAs, to then inhibit protein translation, is thus far their only recognized function. However, considering the carrier effects, it is clear that functional alterations mediated by exosome transferred miRNAs do not entirely act in this conventional manner. To strictly apply the hormone concept, where the molarity of the transferred miRNAs is just considered alone, excluding the likelihood that they have intracellular amplifying mechanisms due to their exosome carriage is a denial of the actual biology. Thus, as noted, exosome extracellular and intracellular carrier actions, which are the physiological mode miRNA transfers provide additional mechanisms with, enable enhancement of their conventional miRNA effects.

Other molecular miRNA effects beyond mRNA silencing have been considered, but are not yet fully realized. An example beyond mere mRNA targeting of miRNAs involves the emerging information of miRNA acting in the nucleus. These non-canonical nuclear actions of miRNAs include the influence on transcription and other intra nuclear mechanisms of gene regulation. These include silencing or activating transcription via specific gene promoters, and modulating co-transcriptional alternative splicing [5,6]. Such nuclear effects are beyond the canonical concepts of transferred miRNAs acting solely on cytoplasmic mRNA [1,2,7,8]. Furthermore, a recent study found nuclear miRNA interacting with DNA via the RNA stem-loop acting on sense and antisense DNA strands with suppression of transcription [9]. Additionally, miRNAs have been shown to interact at the genomic loci, where enhancer-derived RNA are transcribed, and can increase the mRNA levels in adjacent genes by promoting a transcriptionally active chromatin state while altering alternative splicing profiles [10].

Relevant to conventional ideas pertaining to hormones or cytokines, the measured total miRNA concentration in plasma is in the range of 2–6 fentomoles per milliliter [11]. A single miRNA type, thus way below femtomoles among this mixture, could be crucial for a given function, or a few miRNAs may act in concert. This is therefore only a small fraction of the total miRNA amount in plasma, that we in fact found can be sufficient when carried by exosomes [12] (Figure 6b). Thus, it seems that if an individual circulating extracellular miRNA is functional, then when acting alone they would be functional at levels that are likely to be far below femtomoles. This is another reason to recognize an important role for the augmenting exosome carrier effects on the established biological functions of the miRNA delivered to the target cells.

The carrier augmentation of the effective concentration of low-levels of free miRNAs was discussed in the prior paper [13]. We pointed out that adding say 10 femtomoles of miRNA to just a milliliter of plasma results in a concentration of 10 femtomoles per milliliter (10^−3^ L). In contrast, considering that instead the 10 femtomoles are in the 10 × 10^10^ exosomes normally found per milliliter of plasma, with each exosome at an average diameter of 100 nano Meters, having a calculated internal volume of 50 × 10^−18^ L (attoliters), this results in a concentration of these miRNAs of only 10 femtomoles per 10^−8^ L, not per 10^−3^ L (i.e., 10^10^ exosomes × 10^−18^ L = 10^−8^ L). Therefore, exosome carriage brings a much greater concentration; here a 10^5^ = 100,000 fold increased concentration (10^−3^ to 10^−8^ L); i.e., from a femto molar concentration to a 100 nano-molar concentration (from 10^−15^ Moles or femtomoles to 10^−10^ Moles or nano-molar); thereby constituting an obvious carrier-acting effect, with EVs delivering a much higher concentration, likely with greatly enhanced biologic function as a result of the miRNA transport being in a very tiny volume of exosomes [13].

In fact, although the major proportion of circulating miRNAs is in exosomes [14,15,16,17,18], certainly some are free and protected from extracellular RNases by attachment to chaperones, like *Argonauts* [19,20,21], and lipoproteins, and in these forms they are also able to be transferred to targeted cells [22,23]. miRNAs not associated with exosomes, but instead borne by such chaperones, seem to function via an alternate pathway of miRNA action. However, in fact, this finally can involve their subsequent in vivo association with exosomes for later delivery by carrier mechanisms via locally present and relevant exosomes [12]. Therefore, these resulting exosome carrier-acting effects can also apply to these original non-exosomal modes of delivery.

In contrast to miRNAs, specific hormones like steroids are at nano-mole (10^−9^) amounts without carrier delivery mechanisms [23], compared to some experiments showing truly functional effects of exosome-delivered femtomoles (10^−15^) of miRNA [12] (Figure 6b). Therefore, considering just miRNA separate from its delivery via exosomes, their activity would be mediated at more than a million fold lower amounts than hormones and cytokines (i.e., 10^−15^ femtomoles of miRNA compared to nano-mole (10^−9^) amounts of hormones and cytokines. Further, peptide hormones like parathormone and some cytokines, like IL-4 and IL-6, are normally present in the blood at pico-molar concentrations (10^−12^), but these blood levels can increase 1000-fold with some forms of cell stimulation [24]; still suggesting that in some instances separate miRNAs can act at much lower levels [i.e., about 10^−15^ moles or 100,000 (10^5^) fold less] when carried by exosomes [12] (Figure 6b).

We have noted that the conventional hormone *stoichiometry* formulation for intracellular activity of miRNA *estimates* that intracellular levels of a given miRNA need to be at about 100 to 1000 miRNA copies per targeted cell for standard effects due to interacting with mRNA [25,26,27,28,29,30,31,32,33]. Therefore, in the absence of known cellular surface “receptors” for miRNA, we hold that it is unlikely that plasma miRNA at femtomolar concentrations can function like hormones. Accordingly, we hypothesize that the assumption of miRNAs acting like hormones in a canonical fashion is not appropriate. Therefore, since miRNAs are in EV like exosomes, we have proposed instead that they deliver mRNA silencing by “exosome carrier effects”; bringing many possible new properties to miRNA effects, as reviewed here and elsewhere [34]. In fact, without specific receptors and considering the non-physiological nature of direct addition isolated miRNAs or artificial genetic expression of miRNAs; both involving artificial non-physiological laboratory experiments, it seems that delivery by carrier exosomes may be the only mechanism for intercellular transfers of miRNAs.

## 4. Exosome Surface Activation of Target Cells Are Carrier Effects

For exosomes, and other EV like larger MV, a key step toward transferring function to targeted acceptor cells, is the binding and then fusion of their mutual surface constituents. These consist of complex distinctive and unique target cell binding surface molecules on EVs consisting of potential ligands like proteins, lipids and poly saccharides, with the targeted cells plasma membranes having corresponding semi-specific surface receptive binding molecular arrays [13,35]. Thus, carrier effects can involve the binding of exosome surface signature membrane proteins to appropriate matching receptors on the target cells [36,37,38], and subsequent exosome uptake mechanisms that can vary depending on the recipient cells involved [35,36,39]. Exosome surface ligand molecules that are potential target cell activators include: heat shock proteins (HSP) [40,41]; heparan sulfate [42]; survivin [43]; integrins [44]; lactadherin [45]; intercellular adhesion molecules like ICAM-1 [46]; KIT-ligand for c-KIT receptor (CD117) [47]; fibronectin [48]; fas ligand [49]; epidermal growth factor receptor (EGFR) [50]; amphiregulin [51]; TLR3 and TLR4 [52]; signaling molecules like wnt [53,54] and Ephrin [55]; cytokines like surface-bound TGF-b [56,57]; and IFN-g and its receptor [58]; as well as common membrane receptors for transferrin [59] and insulin [60].

Other demonstrated potential exosome surface activators of targeted cells include: antibody (Ab) free light chains [61,62,63,64]; *αβ*-T cell Ag/MHC receptors [65,66]; procoagulants [67,68]; tetraspanins [69,70]; TIM4-binding phosphatidyl serine [71]; heparin-binding proteins [72]; histones; and annexins [73,74]; MHC class I and II; immune checkpoint or negative co-stimulation molecules (PD1, PDL-1) [75,76]; complement components [77,78]; glycosaminoglycans and proteoglycans [79]; binders of the TAM family of tyrosine kinases [80]; enzymes like ADAM family members [81]; sialidases [82,83,84]; DNA [85,86,87]; various lipoproteins like Apo E [78]; LDL receptor [88]; tumor necrosis factor receptor-1 [89,90] and various thiols.

Overall, this is a staggeringly enormous list of possible exosome surface entities that might activate the targeted cells, to promote consequential intracellular transfers of miRNAs; perhaps therefore augmenting effects of low doses of the exosome carrier transferred miRNAs. Furthermore, there is great heterogeneity here since exosome surface marker protein expression is different even among related cell lines and likely even from a single cell [91,92]. These surface proteins influence the uptake rate into the recipient cells; according to their particular expression on exosome surfaces [93]. Therefore, it is likely that different membrane protein expression and expression of particular arrays on exosomes likely play a role in tissue-selective and cell-selective uptake. Additionally, further target cell activating exosome carrier effects can be mediated via yet other neighboring exosomes that are also impinging on the target cell surface, but not eventually transferring miRNAs.

Note that with cell activation, endogenous copies of a given miRNA in the targeted cell can vary six-fold per cell, and the total RNA can vary ten-fold to influence the effects of the transferred miRNAs [94]. In addition, with certain cell activations and the resulting cell differentiation towards greater maturity, there can be up to a progressive six-fold increase in levels of a particular miRNA relative to the total RNA; that can at least be a 10-fold rise [95,96]. Thus, the relative concentration of a given miRNA carried by an exosome for mediation of biological effects might relate functionally to varying endogenous cytoplasmic levels, dependent in part on such activation of the targeted cell [95,96]. Therefore, a small amount of a given transferred miRNA can possibly go a long way. Thus, when very little is added to a lot of endogenous miRNA, levels may reach a threshold for productive interactions with particular cytoplasmic mRNA, in order to achieve exosome-carrier mediated epigenetic functional effects.

It is useful to keep in mind that each single exosome has numerous surface molecules, able to interact with a variety of different cognate receptors or ligands on the plasma membrane of the targeted acceptor cells, and which can then, after uptake, interact additionally with the endosome membranes of recipient cells. From the point of view of exosome carrier effects, these sets of numerous interacting and possibly activating membrane interaction pairs mean that exosomes are likely to display complex cell-binding and possible cell activation kinetics. These interactions therefore transmit multiple signals of variable intensity, or even for other neighboring exosomes, activate the targeted cells without their being taken up. Considering carrier effects, these cognate binding interactions lead to possible multiple mechanisms of exosome mediated target cell activation, and possible fusion of the incoming exosomes. Subsequently, various exosome mediated intra cellular activating mechanisms that may influence pathways and processes triggered for eventual functional effects of the transferred miRNAs and other exosome delivered cargo must also be considered. It is further relevant to note that, the human genome encodes more than twenty possibly involved activatable endosomal binding or signaling proteins; some with known fusogenic abilities [97,98].

One example from our work is in the healing treatment of spinal cord injury (SCI) with mesenchymal stromal cell (MSC)-derived exosomes. In this case, a particular subset of these vesicles specifically targets a subset of M2-type “healing” macrophages among many inflammatory cells at the local SCI site. This is observed to be far above any targeting of many surrounding M1-type pro-inflammatory macrophages, and also a variety of other leukocytes and tissue neuronal cells; such as macrophage related microglia, that are not targeted [99]. When these (MSC)-derived nano-vesicles are synthesized in donor cells, surface membrane proteins of the exosomes are, structurally, arranged differently compared to the donor cell membranes. Here, the hydrophobic sequences of some exosome surface proteins are reversed from conventional cell surface membrane orientation, such that they are free to insert into the target cell plasma membranes, so that they undergo receptive lipid reorganization, and protein restructuring [100]. Together this can mediate target cell fusion with single vesicles such as particularly activated exosomes that are thus far the only currently demonstrated process of vesicle/cell interactions [37]. After these processes of intricate and diverse binding to the surface of target cells, the impinging exosomes are taken up intracellularly by phagocytosis [101,102] or micropinocytosis [103,104], to then move into endosomal spaces with clathrin coated membranes, or via lipid rafts [105], to then proceed on into cytoplasmic phagolysosomes, or perinuclear lysosomes [106].

## 5. Exosome Resistance to Digestion in Phagolysosomes Is a Carrier Effect

It is quite crucial that subpopulations of “activated” exosomes with altered membrane lipids, are known to resist very low gastric pH with mixed digestive enzymes [107]. Thus, such subsets should be able to resist these conditions present in phagolysosomes that generally have a similar mixed acid/enzyme environment [108,109,110,111,112,113,114,115,116]. Perhaps as a consequence, many current studies employing confocal laser scanning fluorescence antibody microscopy show that labeled exosomes are frequently noted in lysosomes or phagolysosomes of the targeted cells. The exosomes in this usually noxious and destructive environment seem to be intact [102,106,107,117,118,119,120,121,122,123,124,125,126,127]. This suggests that there may be distinct new mechanisms of the intracellular release of functional molecules contained in the exosomes, like miRNAs preserved in these intracellular digestive vacuoles, mediated by yet unknown mechanisms to affect EV carrier assisted targeted cell intracellular functions. Observed slow drifting diffusion of exosome contents in local cytoplasmic microenvironments is consistent with such a previously unrecognized alternate intra cellular release mechanism [106,128].

Therefore, it is hypothesized that the release of contents like miRNAs from intact exosomes within these intracellular digestive vacuoles may be via an alternate release process that may, in one postulated scenario, be low-dose-over-time. This is in contrast to the conventional phagocytic uptake of bacteria.

In this case, there is rapid dissolution of the organisms in the phagolysosomes; enabling the immediate and total release, as well as the likely digestion of, bacterial contents. The possible low-dose-over-time intracellular release of miRNA from seemingly intact exosomes may have previously unexplored biochemical properties that can account for an important carrier effect.

Of course, it has not yet been determined that those visualized exosomes are a subpopulation that are not able to immediately release their contents and thus are still detected, but eventually will release their miRNA. In contrast, those having already released their contents may have dissolved and therefore cannot be detected by these methods. Alternately, those EV visualized as intact in non-releasing exosomes that may have activated pathways are important as effects of the miRNAs already released by other exosomes that can no longer be visualized.

## 6. Systems Biology Is More Appropriate for Delivered Exosome Functional Mirna Responses Than Traditional Stoichiometry

As noted above, exosome functional responses mediated by miRNA transfers likely involve consideration of multiple possible carrier effects. Therefore, these functional extracellular and then intracellular carrier mediated aspects are far more complex than simple biochemical reactions due to added miRNA alone, or genetic expression; both usually analyzed by conventional Michaelis–Menten rules, without a thought of the exosome carrier aspects. This classical analysis of biochemical reactions originally concerned the product of an enzymatic reaction that depended on concentrations of the substrates. Such formulations are appropriate to reductionist thinking for single substances alone; like a hormone or a cytokine and its receptor [129,130,131], as we argued above, is not appropriate for exosome transfers of miRNAs. We propose that such simple stoichiometry does not apply for functional responses delivered by exosome miRNAs that are accompanied and influenced by numerous important vesicle mediated carrier effects.

Note that reductionist stoichiometry concepts even hold that complex entities can be understood by this analysis of individual components, that are then brought together to understand the whole [132,133]. However, it is increasingly evident that this approach is insufficient for understanding a process like exosome transfer of miRNA epigenetic functions. Instead these processes may be analyzed more comprehensively by applying concepts of systems biology [134,135,136,137,138,139]. Thus, we hypothesize that such “big thinking” alternative convergent systems’ biologic concepts apply to the functional activities of miRNAs delivered into targeted cells by exosomes. Systems biology is a more appropriate deductive approach that brings divergent components together to create novel synthesized “out of the usual box” formulations that may best be analyzed as an interacting whole.

## 7. Kinetics of Exosome Carrier Influences for Analysis of “Real Life” Four-Dimensional Effects

Analysis by systems principles can lead to previously unimagined ideas and concepts, generating new experimental approaches to establish unanticipated algorithms. Systems biology would hold that exosome transfers of miRNA functions are composed of inseparable complex interacting components that we posit are significantly guided by exosome carrier effects. Such an analysis involves the inter-disciplinary study of complex interactions within a such a diverse biologic system, using a new holistic approach to identify processes and not merely just the participating components [140,141,142,143]. A significant dynamic kinetic influence on all the above arguments concerning carrier effects on the exosome mediated transfers of miRNAs is that the involved exosomes likely bind to the target cell surface over time and then can similarly act dynamically in the intracellular processes [52].

Finally, there likely are many exosomes that are impinging on the targeted cells, and perhaps partially interacting with their surface without full binding or uptake, but still possibly influencing intracellular events just by their partial surface activation of the target cells [144].

**Figure 1:**
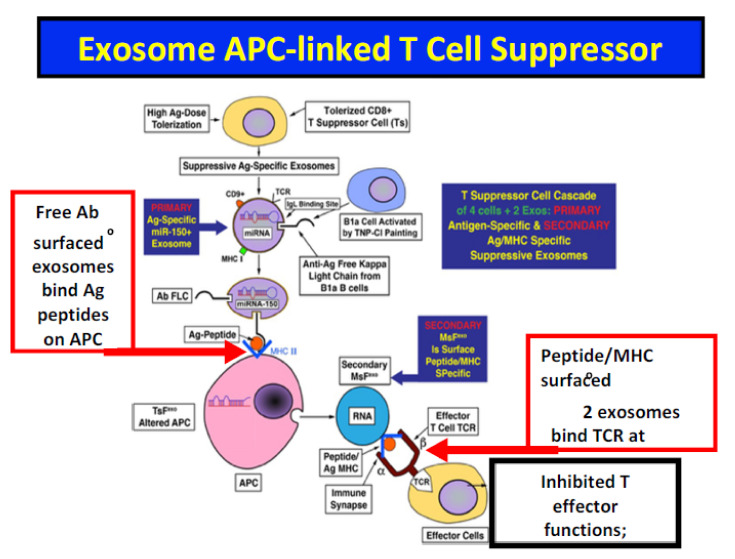
Exosome APC-Linked T Suppressor Circuit in Convalescent Plasma; Demonstration of the Primary and Secondary Varieties of Ag-Specific Exosomes.

For example, in our studies of suppressive systemically administered exosomes modulating classical cutaneous delayed-type hypersensitivity (DTH) immune responses, effects of administered immunoregulatory T cell and B cell-derived exosomes on the time course of elicited DTH responses were determined. It was uncovered that the dynamically measured DTH responses are modulated by systemic administration of a single physiological transfer dose of Ag-specific suppressive exosomes delivering miRNA-150 in particular. This single exosome carrier delivered miRNA strongly inhibits a complex multicellular and multi mediator Ag-specific immune inflammatory response in the ear skin of recipients over the next four–five days, resulting in 80–95% suppression of individual responses measured daily for the subsequent four days [60,61,62,63]. In fact, there is a linear immunomodulatory circuit, in which the miRNA-150 transferred by the Ag-specific Ab targeting of the primary (1°) suppressive T cell-derived exosomes induce the Ag presenting cells (APC) to be altered over several hours to then release different secondary (2°) suppressive exosomes that produce and then release yet another miRNA to inhibit the effector T cells by acting at the central immune synapse [62] (Figure 1). Such dynamic complexity consisting of many steps applies to almost all living systems, in which their biologic behavioral changes certainly include such kinetic changes over time, and require analysis in four dimensions; i.e., length, width, depth, and time. This makes responses really impossible to analyze just from the static properties of individual or even some combined parts by the two dimensional stoichiometry of the Michaelis–Menten rules.

Murine donors are treated with high systemic doses of Ag over time to mimic exposure to the viral Ag during acute COVID-19 infection. This induces in the plasma suppressive primary (1°) exosomes derived from CD8-pos non-Treg suppressor T cells that become Ag-specific by surface expression of Ab free light chains derived from companion B1a B cells that also were activated by exposure to excessive viral Ag. The Ab free light chains bind Ag-peptides in MHC on the surface of APCs to alter them epigenetically via the exosome transfer of their contained miRNA-150. These functionally altered APC can then produce secondary (2°) suppressive exosomes with surface expression of the Ag-peptide/MHC complexes that can conjugate bind to the αβ-TCR of effector T cells at the immune synapse to suppress their functions. This results in transfer of other miRNA to inhibit effector T cell production of INF-**γ** and other tissue altering cytokines.

Therefore, exosomes are considered to be secreted subcellular organelles composed of many separate entities that function together in targeted cells over time. For impingement and binding on particularly receptive targeted cells, one must consider that the biologic effects are due to perhaps millions of individual varied complex nanoparticles with quite variable surface phenotypes, like EVs such as exosomes and MV, potentially acting dynamically over time. This can be illustrated as multiple different interactomes that are not in standard flat two dimensions, and not even in three dimensions. Additionally, analysis should also include accounting for not only RNA sequence but also for three-dimensional arrangement of the miRNA molecules in space [145], resulting in interactions that can produce functional effects that can be augmenting or inhibiting while acting at variable strengths.

To this analysis we must further add the dynamic responses over time of the targeted cells to the involved transferred entities. Such dynamic kinetic aspects of three dimensional interactions become therefore a fourth dimension of intracellular interactome targeted cell genetic sequence communication that is induced by the carrier mediating exosomes [144,145,146,147,148,149]. This represents new sets of exosome-mediated molecular interactions, often dominated by their constituent multiple coding and non-coding transferred RNA types, and also their protein cargo of enzymes and signaling molecules and lipid cargo, ongoing together in eligible semi-specifically receptive targeted cells, akin to established networks of signaling and metabolic pathways recognized in other systems.

In these early times, there have been only a few published studies considering systems approaches to exosomes [150,151,152]. Naturally there have been more numerous systems biology descriptions proposed for the immune system [146,153,154,155,156,157,158,159] and also for a variety of subjects that are related to exosome actions [160,161,162,163,164,165,166].

We need to begin to understand these merged functional kinetic interactome systems, in which exosome miRNA is transferred and that depend on multiple EV mediated carrier aspects, inducing detected and quantitated functional consequences, altering biologic actions and relationships between and among various cell types. Such affected cells are present in a tissue neighborhood that features a cloud of exosomes from diverse local and distant cells, occurring in the extracellular space between all of the potentially interacting cell types of that particular microenvironment [167]. We will need to examine how the individual components dynamically interact as they operate over time.

Such analysis requires, first and foremost, robust functional assays. These must be objective, quantitative and highly reproducible. The functional assays need to be very rigorous, since at this stage the input exosomes are very heterogeneous mixtures of different types of density gradient validated nano vesicles that can include exomeres, exosomes, MV, and also amphisomes and apoptotic bodies, Therefore, the carrier effects are numerous and the transferred molecules are quite diverse.

The hope is to become able to construct models of exosome carrier promoted effects on the targeted cells; including delivery, intracellular release and functional actions of miRNAs over time, that are analyzed by systems approaches. This should enable the discovery of holistic exosome miRNA mediated complex biologic entities and processes. This would be akin to the study of organisms and tissues, not merely molecules and their receptors.

## 8. Analysis of Ag-Specific 1° and 2° Suppressive Exosomes Acting in Series on Contact and Delayed Sensitivity

As noted, when examining exosome carrier-assisted functional miRNA transfers, initial determinations must include repeated blinded objective observations of robust quantitative altered functions of the targeted cell functions modulated by the transferring exosomes, including in a major way, actual molecular epigenetic effects of the transferred miRNAs. Furthermore, as a fundamental, it is also required to precisely determine the time course and dose response of the functional effects delivered by the carrier-acting exosomes. Dose response and time course studies are essential and basic to physiology, but are not often conducted in exosome studies. These required initial data sets can then enable the beginning of the analysis of processes for conceiving further optimal experiments that can uncover unanticipated aspects of the process under study. In our case of exosomes mediating Ag-specific in vivo T cell suppression of effector T cell immune inflammatory responses of the CS type of DTH to low molecular reactive hapten Ag, our first paper uncovered the particular importance of miRNA-150 transfer, and importantly determined exosome dose response and time course by both in vivo and in vitro quantitative assays [63]. These findings uncovered an unexpected therapeutic duration of at least four days for a single systemic physiologic dose of suppressive exosomes, administered once at the peak of the recipient DTH response. Our second paper, again in the hapten Ag CS system of DTH, tested the dose response of miRNA-150 association with a given physiologic number of 10^10^ Ag-specific B1a cell-derived exosomes; surprisingly, this showed that a remarkably low dose of 50 femtomoles of miRNA-150 was sufficient for the systemic functional effects in the ear skin of recipient mice undergoing active sensitized immune responses [12] (Figure 6b).

In the third and fourth papers, now in classical protein induced DTH responses [60,62], we established that the exosome surface Ab bound to Ag-peptides complexed in MHC on the surface of affected APC [60]. The delivery of miRNA-150 induced the targeted APC to produce secondary (2°) suppressive exosomes that had surface Ag-peptide/MHC for the regulation of the function by finally targeting DTH effector T cells in cognate binding via their αβ-TCR at the immune synapse by the transfer of separate different miRNAs [61]. These findings confirmed the above kinetics in different experimental systems, including studies entirely with monoclonal reagents, consisting of classical DTH to the OVA protein Ag ovalbumin in the murine OTII system. These are transgenic mice that express monoclonal alpha and beta-chains of TCR specific for ovalbumin 323–339 peptide in the context of MHC class II Ag. We showed that systemic treatment with immunosuppressive exosomes, that was begun at the height of the elicited DTH response was inhibited over the subsequent 4–5 days, even when the exosomes were administered orally [60,62].

Further controls are needed to begin towards fully accomplishing a more comprehensive and accurate analysis of exosome mediated transfers of functional miRNAs. These must be carefully designed in order to attempt to prove that the results and derived concepts might actually be wrong.

If correct, there then needs to be determination of particularly involved miRNA from the secondary (2°) suppressive exosomes, its molecular target and the mechanism of the actual exosome carrier-delivering and carrier-acting effects on the receptive targeted cells. This will likely involve variations in methods, elucidating the mechanism of targeting depending on the surface signature of the exosomes and the corresponding molecular array on particularly receptive recipient cells and, importantly; whether the essential findings can be reproduced in another biologic system. These experiments then will lead to determining dynamic interactions, the identification of targeted intracellular cell biologic and biochemical networks and then the encoding DNA and resulting functional alterations. This will likely involve the identification of targeted transcription factors that are affected by the exosome-transferred miRNAs, appropriate affected DNA sequences and consequent altered specific gene expression leading to modified function; all with current technologies that will be a major undertaking.

From such studies, there should then emerge a comprehensive database to undergo the meta-analysis for beginning the formulation of algorithms with expression profiling and clustering analysis to identify altered expression of genes of known function [168]. Posttranslational mechanisms of regulation can then become available to be incorporated as large-scale data, hopefully via high throughput assays to incorporate all the data. This is needed to formulate new hypotheses, and to then generate more refined algorithms to hopefully suggest experiments that reduce the number of ambiguous network hypotheses. At present, such algorithms are far from reaching a level of practical application in the functional exosome field. However, early drafts to be progressively refined will be useful for determining the optimal order and nature of experiments needed to resolve ambiguous results by eventual hypothesis-driven research.

In summary, systems biology is based on understanding that the whole is greater than the sum of the parts. It is collaborative, by integrating many scientific disciplines to predict how a system changes over time and under varying conditions aimed at designing multiscale models. Systems biology here would examine the living entity of exosome transfer of miRNA-mediated epigenetic functional effects in targeted cells. This is seen as a multitude of simultaneous interactions at various levels occurring together at any one time, combined to emphasize the importance of the whole, via discerning interdependence of its parts and kinetic aspects, compared to the standard reductionist approach of dealing with isolated components in two dimensions.

As an example, reductionist approach studies of just the molecular biochemical effects of exosome transferred miRNA alone on mRNA translation are often conduced with luciferase assays that exclude any alternative mechanisms beyond the effects of miRNA transfers on mRNA translation. Instead, in systems analysis this simple aspect is considered, along with multiple levels of varying associated molecular and cellular activity that are pooled for analysis together. Such a systems biology approach to exosome miRNA transfers is interdisciplinary research, requiring technologies and expertise from a spectrum of scientific disciplines and indeed is beginning to be applied to the analysis of exosome function [169,170,171,172,173,174,175,176,177,178,179] now clarified further with CRISPR technology to more easily use mutations for analysis of the central alterations on gene expression [180,181,182,183].

## 9. Exosome Carrier Effects of Diet Absorbed Maize microRNAs in Pigs

An example is a very thorough recent study of pigs ingesting dietary corn for seven days, in which it was shown that the maize-derived miRNAs were transported into porcine tissues in a subset of exosomes that were likely activated. These EV partially resisted harsh cooking (steaming), which might be due to the lipid composition of their special carrier surface. These orally administered exosomes passed stomach digestion in vivo, and showed similar resistance in an in vitro model of the upper GI tract [109].

They demonstrated cross-kingdom miRNA transfers. Thus, after the ingestion of corn, a selected group of plant-derived miRNAs were found in various porcine tissues and in serum from their systemic circulation. Furthermore, the plant miRNAs in the serum were in host exosomes, suggesting that after surviving the stomach, the plant exosome associated miRNAs were placed into host exosomes.

These plant miRNAs, now in mammalian exosomes after oral administration, were molecular chemically verified as plant in origin. Many miRNA sequences are universal through all life forms and these miRNAs could be in this group. However, it is well known that miRNAs from plants are much more sensitive to oxidation, compared to mammalian miRNAs as a result of unique chemical conjugations. Accordingly, the miRNAs in the tissues and blood of the pigs that were thought to be of plant origin were confirmed by showing that they were significantly more sensitive to oxidation compared to those of the host [109].

Finally, their transfer to the host, after passing the toxicities of the stomach, were demonstrated to induce functional changes after systemic absorption. The plant miRNAs from ingested corn detected in these mammalian pigs were shown to significantly increase appropriate host mRNA levels; both in vivo and in vitro. This suggested that these plant miRNAs were able to alter function in targeted cells in the pigs [109]. Overall, these strong data suggest that exosome carrier effects of plant associated dietary miRNAs can become present in host mammalian exosomes to alter targeted host mRNA levels; thus they are potentially able to affect the function of targeted genes. Note therefore that the proposed carrier-delivery and carrier acting processes occurring in sequence occurred in this pertinent example. It seemed likely that plant miRNA in subsets of plant exosomes were able to pass the harsh acid-enzymes of the stomach, and then become associated with the host exosomes that similarly are able to resist blood RNases to then be active in the host target cells that are receptive to these EV, leading to the mediation of epigenetic functional effects.

These results are relevant to the initial prescient and still controversial studies from the laboratory of Chen Yu Zheng in Nanking China. They showed the transfer of dietary plant (rice) miRNAs in humans, that then were reported to enter host exosomes to then go into the systemic circulation and subsequently affect liver cell mRNA levels and associated biochemical function [184]. These important disputed results can now be viewed in the context of carrier effects of exosome subpopulations. In general, pertaining to this crucial area, aspects of the ingestion and perhaps background effects produced by other processes present in the GI tract, such as inflammation or drug treatments, may produce activated GI cell-derived exosomes that eventually provide their carrier- delivering and carrier-acting effects, allowing the targeting of particular recipient liver cells to make possible transfers of minute amounts of specific miRNAs [185]. Analogous changes from other processes generating activated exosomes may also pertain to some of the other published studies of cross species and cross kingdom exosome mediated functional miRNA transfers [186,187,188,189].

Furthermore, and pertinent here, there has been some concern by a few investigators about such cross-kingdom transfers of miRNA via exosome delivery and subsequent biologic actions [190,191,192,193,194,195,196]. These negative data and assertions beyond the data have resulted in detailed and cogent replies from the original investigators who studied the effects of a chronic rice diet [184]. In a subsequent study of humans acutely ingesting watermelon juice, featuring more developed techniques to measure minute amounts of plant-derived miRNAs amid huge excesses of similar host miRNAs, had very similar findings [197]. In newer work they showed the therapeutic potential of cross kingdom plant miRNAs in humans with hepatitis infections [198]. Finally, the wide potential biologic, clinical and disease significance of such cross-kingdom transfer of genetic material has since received thoughtful reviews and has been supported by additional favorable data in many studies by other investigators [109,197,199,200,201,202,203,204,205,206].

## 10. Positive and Negative Data of Plant miRNAs Effects in Mammals Are Likely Both Correct

I have had extensive personal experience regarding such controversies concerning the original work in my laboratory compared to the negative studies in the work of others. In two different cases these differences were aired in public at prominent meetings with large audiences; at which the combatants were convinced that they were right. Indeed, it is likely that they both were right. The experiments in these past controversies showed variations in results in what appeared to be similar systems.

However, it was eventually discovered that small differences in protocols or interpretations of the data led to very different conclusions about the results. In one instance, differences in the immune dose response to very similar antigens at different effective doses in two different genetic strains of mice was the cause of opposing results; with both therefore being correct [207,208].

In the current controversy about the effects of dietary plant-derived exosomal miRNAs with biological effects in recipient mammalian hosts, there are instead vast differences in tested systems, reagents employed and measurements of results to account for different results obtained. Furthermore, there has been little interest or enthusiasm in trying to exactly repeat all of the aspects of the particular experiments, in the laboratories claiming divergent data in response to the published findings of others, such as the original chronic rice ingestion studies mentioned above [184]. Additionally, there has been little realization that in these systems per se, and especially concerning low levels of transferred miRNAs, that there can be wide qualitative and quantitative carrier effects of the exosomes, as well as host cell receptivity for particular exosome subsets, their miRNA delivery, subsequent intracellular biological processes and effects, and variation in reactivity.

This is to be expected, since currently employed systems of study are biological and very complex, compared to simple merely chemical data with suitability for a Michaelis–Menten-type stoichiometric analysis. In many conflicting cases, it is falsely proposed that if positive results in system A of other investigators are correct, then should any attempt at system B, based on similar ideas but in very different systems, turn out to be negative, then the results of A must be wrong. This is very poor experimental science.

However, results suggesting that some of the experiments are flawed due to artefactual reagents being employed, or human or murine host miRNAs being misinterpreted as plant miRNAs, or other inconsistencies, must be carefully dealt with [192,193,209,210,211,212].

## 11. Low Exosome miRNA Levels Mediate Function via Carrier Aspects

Our particular experiments in exosome mediated suppression of DTH determined that, at least in some systems, low levels of miRNA can be carrier-delivered and have carrier-acting functional effects in vivo and in vitro [12,60,61,62,63]. This was determined via rarely reported dose response studies of miRNA association with B suppressor cell exosomes that were able to regulate classical T effector cell-mediated cutaneous contact sensitivity (CS) immune inflammatory responses to hapten Ag in vitro and in the skin in vivo [12,60,61,62,63]. These studies definitively demonstrated that these B cells that were imitating CD8^pos^ T cell-derived suppressive exosomes, delivered the pertinent miRNA-150 to targeted cells at an in vivo functional end point dilution of only 50 femtomoles (750 pg, 10^−15^ Moles) [12] (Figure 6b).

In a recent follow up study of the T cell regulation of protein-induced immune classical delayed-type hypersensitivity (DTH), analogous suppressive exosomes delivered the pertinent miRNA-150 to targeted cells [60]. Here, an analogous dose response experiment showed significant in vivo suppression of DTH responses to an end point dilution of a far greater amount of a thousand-fold more B cell exosome associated of this same pertinent miRNA-150 (300 ng, 10^−13^ Moles). Therefore, this was quantitatively very different from hapten-Ag CS, which is a very different system [12,60].

These data in CS and DTH verify an aspect of the dietary claims for oral route delivery, achieving systemic activity of such small amounts of miRNA delivered by exosomes. They also show the wide variations in functional miRNA carried by exosomes in different but analogous systems; echoing the wide levels of plant miRNA copies found in human tissues in the original paper on chronic rice ingesting Chinese individuals [184]. Our data further demonstrated that in addition to IV and IP administration of these DTH-suppressive exosomes, that small amounts of exosomal miRNA also can transfer the systemic inhibitory function of just miRNA-150 among their diverse cargo over at least four days after a single administration of a physiologic dose. In fact, these recent studies of suppression of protein Ag DTH have shown that the oral route of administration of the suppressive exosomes is as good as, and even better than, the systemic IV or IP routes [60,62]. This finding favors the idea that in some instances dietary food might provide miRNA to actually systemically act functionally in vivo after oral administration [12,60,62], as originally proposed in the rice dietary studies [184].

## 12. Exosome-Carried miRNAs May Add to Already Proceeding Intracellular Pathways

The most important aspect of the rice diet/miRNA stimulated controversy is the functional significance of the reported low amounts of miRNA. Hanging over these issues are separate unsettled complexities of the miRNA field about genetic mechanisms underlying unknown and confusing canonical functions. There is uncertainty on the point of how multiple miRNAs can have effects on many separate mRNAs [213], and the reverse; that each mRNA can supposedly be acted on by multiple miRNAs [214]. Another unsettled issue is the possibility that miRNA can act via non-canonical pathways. A developing possibility is that there are functions of miRNAs beyond the cytoplasm, occurring in the nucleus acting on transcription and other DNA gene regulatory processes [215].

In fact, growing evidence suggests cytoplasmic mechanisms of miRNA actions beyond mRNA silencing. As most fields develop, it comes to be recognized that there are alternate non-canonical pathways, that here could involve exosome carrier effects on miRNA functional actions. This seems likely, given that miRNAs and resistant EV like exosomes have been evolutionarily present for billions of years [13]. Other postulated cytoplasmic roles of exosome transferred miRNAs include: re-thinking miRNA-mRNA interactions concerning differences between the binding to mRNAs and the actual induced functional changes [214,216]; or competing endogenous RNAs (ceRNA) that might inhibit the effects of target cell endogenous miRNAs, perhaps acting as an miRNA sponge. These competing endogenous RNAs could combine with miRNA carried by the exosomes, to then tilt toward function due to the very small added amounts of exogenous exosomal miRNA. Therefore, competitive action might enable a threshold to be reached.

Furthermore, there likely will be great variability according to the biological or clinical context or conditions of the pertinent postulated exosome carrier effects, the resulting miRNA:mRNA interactions [217,218], and the final epigenetic alterations. Note that new results show widespread protein translational effects of miRNA seed sequence matching to mRNAs, so that multiple mRNA seed sites for the same or different exosome carried miRNAs might function cooperatively to give effects on multiple mRNAs [219], suggesting cooperativity between different transferred pertinent miRNAs [220]. Furthermore, there can be both positive and negative effects of miRNAs on transcription depending on some non-canonical pathways [221,222].

Likewise, worthy of consideration are the possible competing effects of the reverse sequence passenger hybridizing strand called miRNA*, that targets five prime conserved ends compared to usual three prime conserved ends of the targeted mRNAs. These could be co-transferred by the exosomes, transferred by other exosomes or already present endogenously [223]. Additionally to be considered, are different effects produced by exosomal carrier transferred miRNA binding to not only the three′ and five′ untranslated ends of mRNA, compared to binding directly to the coding domain of the mRNA sequences. Binding to mixtures of these three sites together or in sequence offer greater possible diversity in the functions of even low doses of exosome carrier transferred miRNAs [224].

## 13. miRNA Two and Three Dimensional Conformations May Affect Intracellular Activities

A potential large effect that has not yet been considered very much is the obvious possible effects on activity of the folding ability of different RNA sequences into unique two and three dimensional formations [225,226,227,228,229,230]. This is analogous to the biologic activity of enzymes or antibodies due to such spatial conformations. Results from three dimensional considerations may provide consequent unexpected mRNA and protein interactions dependent on such three dimensional shape orientations [231], that as noted can possibly imitate the activities of enzymes (i.e., become ribozyme like, etc.) or antibodies. Furthermore, such considerations open the possibility of miRNAs binding target mRNA beyond just the sequence by instead skipping the strict sequence binding according to three dimensional spacings.

These RNA conformations in space are already recognized as having effects on the generation and loading of miRNAs into exosomes in the donor cells. However, expected effects of miRNA and/or mRNA three dimensional conformations on exosome carried transfer of miRNAs have not yet fully emerged [232,233]. Note that when pertinent protein expression is quantitatively considered along with the relevant miRNA:mRNA interactions, there is significantly better correlation with the resulting functional results [234]. Further contributions along these lines could come from newly recognized regulation of gene expression that is due to miRNA molecular target interactions that can reciprocally control the function of the impinging miRNAs [235].

Additionally, there could be quantitative cooperative aspects leading to more diverse and increased cell functional responses due to pertinent transferred miRNA and other RNA-types also carried by the exosomes; like the numerous rRNA and tRNA fragments. This might be via these RNA fragments affecting many mRNAs or other possible miRNA targets. Such interactions would therefore multiply the effects from each delivered miRNA alone on the mRNA seed sites [236,237]; or even mixes of transferred miRNA-3′ and miRNA-5′ cooperative seed sequence effects [219,238]. Although not cooperative in a biochemical and stoichiometric sense, such merging of independent miRNA effects are known to be able to add up to a 25-fold increased repression of mRNA. Thus, such target inhibition can become more sensitive to small changes in abundance of single miRNA sequences, possibly delivered by exosome carrier-acting effects [236,237].

Finally, a recent determination of a possible broad range of miRNA functional sites employed a systemic large-scale transcriptome analysis. Extraordinarily, about two billion potential miRNA interacting mRNA sites in humans were examined [239]. This massive work uncovered not only canonical sites, but several novel non-canonical functional types, that also have been noted previously [239]. Importantly, additional non-canonical sites for miRNA interactions were found to depend on the employed experimental conditions, which is the sort of thing215 that could account for differences in findings with small amounts of miRNA between results of different investigators in the controversy summarized above. This needs to have an exchange of competent lab workers actually doing experiments in each other’s laboratories. The mass findings in this new work had extensive experimental validation by employing multiple arrays and luciferase reporter assays with stringent corrections for multiple testing [239].

The new sites were found to be evolutionary conserved by testing functionally in zebra fish. Most contained Watson–Crick nucleotide pairings at the three′ untranslated site in mRNAs. Although the proficiencies of function based on these new innovative sites were weaker, true function was still induced [239]. These could represent low level augmented stimulation that is mediated by exosome carrier effects on transferred low doses of miRNAs. Overall, these newly recognized expanded interaction site types suggest that miRNA mediation of epigenetic regulation is far more complex than currently thought; especially if considering the added augmenting possibilities brought by consideration of the various effects of the carrier-delivering and carrier-acting effects on functional cellular alterations produced by the involved exosomes.

## 14. Overall Summary

Quantitatively, current canonical rules dictate the minimum copies of an miRNA for functional effects, and thus successful transfers. However, ingested small amounts of xenogeneic plant miRNAs and oral administration of immunosuppressive exosomes exemplify carrier effects on transfers contributing to the delivery and subsequent functional outcome of very low levels of transferred miRNA. These may be mediated by non-canonical minute quantities of miRNAs transferred via carrier effects by exosomes to targeted cells. Therefore, functional low levels of exosome transferred miRNA may not fit conventional reductionist stoichiometric concepts. Accordingly, we have examined traditional stoichiometry vs. a systems biology approach that may be more appropriate for delivered exosome functional responses.

Exosome carrier properties to be additionally considered include: kinetic dynamic aspects of exosome carrier influences that might allow systems analysis of the four-dimensional effects; alternate targets beyond mRNAs; added miRNAs reaching a threshold; three dimensional aspects of the RNAs influencing bindings; and unique intracellular release of exosome carried miRNAs from resistant exosomes in acid/enzyme-rich phagolysosomes. This applied systems approach can then result in a more appropriate understanding of transferred exosome carrier-assisted functional transfers (Table 1).

## 15. Conclusions

The plea here is that the miRNA expert community, perhaps in collaboration with exosome experts, will perform new experiments on molecular and quantitative miRNA functional effects in systems that include exosome delivery, to be varied in type, dose and time to hopefully constitute more appropriate carrier effects and truly current canonical concepts of the consequent miRNA functional transfers by EVs like exosomes.

## Figures and Tables

**Table 1:** Carrier Effects of Exosome miRNA Transfers.

-Resistance to hypoxia and acid/enzyme mixtures
-Attoliter volumes make femto moles miRNA to nano molar
-Surface activating interactions with targeted cells
-miRNA levels reaching a threshold
-Three dimensional aspects of the RNAs
-EV kinetic aspects make transfers four dimensional
-Resistance to intracellular digestion in phagolysosomes
-Primary and secondary circuits of exosome effectors

## Data Availability

Not applicable.

## Abbreviations

Ab	Antibody
Ag	antigen
APC	antigen presenting cells
CS	contact sensitivity
DC	dendritic cells
DTH	delayed-type hypersensitivity
EV	extracellular vesicles
FLC	free light chains of antibodies
GI	gastrointestinal
IP	intraperitoneal administration
IV	intravenous administration
PO	oral administration

## References

[1] Kenneth K.W., Théry C. (2019). Extracellular vesicles or exosomes? On primacy, precision, and popularity influencing a choice of nomenclature. J. Extracell. Vesicles.

[2] Xu J., Camfield R., Gorski S.M. (2018). The interplay between exosomes and autophagy—Partners in crime. J. Cell Sci..

[3] Greenhill C. (2017). Exosomal microRNAs as novel adipokines. Nat. Rev. Genet..

[4] Greenhill C. (2017). Adipose tissue: Exosomal microRNAs—Novel adipokines. Nat. Rev. Endocrinol..

[5] Castel S.E., Martienssen R.A. (2013). RNA interference in the nucleus: Roles for small RNAs in transcription, epigenetics and beyond. Nat. Rev. Genet..

[6] Catalanotto C., Cogoni C., Zardo G. (2016). MicroRNA in Control of Gene Expression: An Overview of Nuclear Functions. Int. J. Mol. Sci..

[7] Liu H., Lei C., He Q., Pan Z., Xiao D., Tao Y. (2018). Nuclear functions of mammalian MicroRNAs in gene regulation, immunity and cancer. Mol. Cancer.

[8] Rappa G., Santos M.F., Green T.M., Karbanová J., Hassler J., Bai Y., Barsky S.H., Corbeil D., Lorico A. (2017). Nuclear transport of cancer extracellular vesicle-derived biomaterials through nuclear envelope invagination-associated late endosomes. Oncotarget.

[9] Miao L., Yao H., Li C., Pu M., Yao X., Yang H., Qi X., Ren J., Wang Y. (2016). A dual inhibition: microRNA-552 suppresses both transcription and translation of cytochrome P450 2E1. Biochim. Biophys. Acta.

[10] Xiao M., Li J., Li W., Wang Y., Wu F., Xi Y., Zhang L., Ding C., Luo H., Li Y. (2017). MicroRNAs activate gene transcription epigenetically as an enhancer trigger. RNA Biol..

[11] Binderup H.G., Madsen J.S., Heegaard N.H.H., Houlind K., Andersen R.F., Brasen C.L. (2018). Quantification of microRNA levels in plasma—Impact of preanalytical and analytical conditions. PLoS ONE.

[12] Bryniarski K., Ptak W., Martin E., Nazimek K., Szczepanik M., Sanak M., Askenase P.W. (2015). Free extracellular miRNA functionally targets cells by transfecting exosomes from their companion cells. PLoS ONE.

[13] Askenase P.W. (2021). Askenase, Exosomes Provide Unappreciated Carrier Effects that Assist Transfers of Their miRNA to Targeted Cells; I. They are “The Elephant in the Room. RNA Biol..

[14] Chevillet J.R., Kang Q., Ruf I.K., Briggs H.A., Vojtech L.N., Hughes S.M., Cheng H.H., Arroyo J.D., Meredith E.K., Gallichotte E.N. (2014). Quantitative and stoichiometric analysis of the microRNA content of exosomes. Proc. Natl. Acad. Sci. USA.

[15] Gallo A., Tandon M., Alevizos I., Illei G.G. (2012). The majority of microRNAs detectable in serum and saliva is concentrated in exosomes. PLoS ONE.

[16] Cheng L., Sun X., Scicluna B.J., Coleman B.M., Hill A.F. (2014). Characterization and deep sequencing analysis of exosomal and non-exosomal miRNA in human urine. Kidney Int..

[17] Cheng L., Sharples R.A., Scicluna B.J., Hill A.F. (2014). Exosomes provide a protective and enriched source of miRNA for biomarker profiling compared to intracellular and cell-free blood. J. Extracell. Vesicles.

[18] Tian F., Shen Y., Chen Z., Li R., Ge Q. (2017). No Significant Difference between Plasma miRNAs and Plasma-Derived Exosomal miRNAs from Healthy People. Biomed. Res. Int..

[19] Arroyo J.D., Chevillet J.R., Kroh E.M., Ruf I.K., Pritchard C.C., Gibson D.F., Mitchell P.S., Bennett C.F., Pogosova-Agadjanyan E.L., Stirewalt D.L. (2011). Argonaute2 complexes carry a population of circulating microRNAs independent of vesicles in human plasma. Proc. Natl. Acad. Sci. USA.

[20] Lv Z., Wei Y., Wang D., Zhang C.Y., Zen K., Li L. (2014). Argonaute 2 in cell-secreted microvesicles guides the function of secreted miRNAs in recipient cells. PLoS ONE.

[21] Li L., Zhu D., Huang L., Zhang J., Bian Z., Chen X., Liu Y., Zhang C.Y., Zen K. (2012). Argonaute 2 complexes selectively protect the circulating microRNAs in cell-secreted microvesicles. PLoS ONE.

[22] Vickers K.C., Palmisano B.T., Shoucri B.M., Shamburek R.D., Remaley A.T. (2011). MicroRNAs are transported in plasma and delivered to recipient cells by high-density lipoproteins. Nat. Cell Biol..

[23] Vickers K.C., Remaley A.T. (2012). Lipid-based carriers of microRNAs and intercellular communication. Curr. Opin. Lipidol..

[24] Kleiner G., Marcuzzi A., Zanin V., Monasta L., Zauli G. (2013). Cytokine levels in the serum of healthy subjects. Mediat. Inflamm..

[25] Landgraf P., Rusu M., Sheridan R., Sewer A., Iovino N., Aravin A., Pfeffer S., Rice A., Kamphorst A.O., Landthaler M. (2007). A mammalian microRNA expression atlas based on small RNA library sequencing. Cell.

[26] Williams Z., Ben-Dov I.Z., Elias R., Mihailovic A., Brown M., Rosenwaks Z., Tuschl T. (2013). Comprehensive profiling of circulating microRNA via small RNA sequencing of cDNA libraries reveals biomarker potential and limitations. Proc. Natl. Acad. Sci. USA.

[27] Brown B.D., Gentner B., Cantore A., Colleoni S., Amendola M., Zingale A., Baccarini A., Lazzari G., Galli C., Naldini L. (2007). Endogenous microRNA can be broadly exploited to regulate transgene expression according to tissue, lineage and differentiation state. Nat. Biotechnol..

[28] Mullokandov G., Baccarini A., Ruzo A., Jayaprakash A.D., Tung N., Israelow B., Evans M.J., Sachidanandam R., Brown B.D. (2012). High-throughput assessment of microRNA activity and function using microRNA sensor and decoy libraries. Nat. Methods.

[29] Landthaler M., Gaidatzis D., Rothballer A., Chen P.Y., Soll S.J., Dinic L., Ojo T., Hafner M., Zavolan M., Tuschl T. (2008). Molecular characterization of human Argonaute-containing ribonucleoprotein complexes and their bound target mRNAs. RNA.

[30] Baccarini A., Chauhan H., Gardner T.J., Jayaprakash A.D., Sachidanandam R., Brown B.D. (2011). Kinetic analysis reveals the fate of a microRNA following target regulation in mammalian cells. Curr. Biol..

[31] Chen C., Ridzon D.A., Broomer A.J., Zhou Z., Lee D.H., Nguyen J.T., Barbisin M., Xu N.L., Mahuvakar V.R., Andersen M.R. (2005). Real-time quantification of microRNAs by stem–loop RT–PCR. Nucleic Acids Res..

[32] Kim D., Sung Y.M., Park J., Kim S., Kim J., Park J., Ha H., Bae J.Y., Kim S., Baek D. (2016). General rules for functional microRNA targeting. Nat. Genet..

[33] Brown B.D., Naldini L. (2009). Exploiting and antagonizing microRNA regulation for therapeutic and experimental applications. Nat. Rev. Genet..

[34] Bissels U., Wild S., Tomiuk S., Holste A., Hafner M., Tuschl T., Bosio A. (2009). Absolute quantification of microRNAs by using a universal reference. RNA.

[35] Buzás E.I., Tóth E.Á., Sódar B.W., Szabó-Taylor K.É. (2018). Molecular interactions at the surface of extracellular vesicles. Semin. Immunopathol..

[36] Buschow S.I., Nolte-’t Hoen E.N., Van Niel G., Pols M.S., Ten Broeke T., Lauwen M., Ossendorp F., Melief C.J., Raposo G., Wubbolts R. (2009). MHC II in dendritic cells is targeted to lysosomes or T cell-induced exosomes via distinct multivesicular body pathways. Traffic.

[37] Prada I., Meldolesi J. (2016). Binding and Fusion of Extracellular Vesicles to the Plasma Membrane of Their Cell Targets. Int. J. Mol. Sci..

[38] Horibe S., Tanahashi T., Kawauchi S., Murakami Y., Rikitake Y. (2018). Mechanism of recipient cell-dependent differences in exosome uptake. BMC Cancer.

[39] McKelvey K.J., Powell K.L., Ashton A.W., Morris J.M., McCracken S.A. (2015). Exosomes: Mechanisms of Uptake. J. Circ. Biomark..

[40] Anand P.K., Anand E., Bleck C.K., Anes E., Griffiths G. (2010). Exosomal Hsp70 induces a pro- inflammatory response to foreign particles including mycobacteria. PLoS ONE.

[41] Gastpar R., Gehrmann M., Bausero M.A., Asea A., Gross C., Schroeder J.A., Multhoff G. (2005). Heat shock protein 70 surface-positive tumor exosomes stimulate migratory and cytolytic activity of natural killer cells. Cancer Res..

[42] Christianson H.C., Svensson K.J., Van Kuppevelt T.H., Li J.P., Belting M. (2013). Cancer cell exosomes depend on cell-surface heparan sulfate proteoglycans for their internalization and functional activity. Proc. Natl. Acad. Sci. USA.

[43] Gonda A., Kabagwira J., Senthil G.N., Bennit H.R.F., Neidigh J.W., Khan S., Wall N.R. (2018). Exosomal survivin facilitates vesicle internalization. Oncotarget.

[44] Fedele C., Singh A., Zerlanko B.J., Iozzo R.V., Languino L.R. (2015). The αvβ6 integrin is transferred intercellularly via exosomes. J. Biol. Chem..

[45] Bliss C.M., Parsons A.J., Nachbagauer R., Hamilton J.R., Cappuccini F., Ulaszewska M., Webber J.P., Clayton A., Hill A.V., Coughlan L. (2020). Targeting Antigen to the Surface of EVs Improves the In Vivo Immunogenicity of Human and Non-human Adenoviral Vaccines in Mice. Mol. Ther. Methods Clin. Devel..

[46] Segura E., Nicco C., Lombard B., Véron P., Raposo G., Batteux F., Amigorena S., Théry C. (2005). ICAM-1 on exosomes from mature dendritic cells is critical for efficient naive T-cell priming. Blood.

[47] Atay S., Banskota S., Crow J., Sethi G., Rink L., Godwin A.K. (2014). Oncogenic KIT-containing exosomes increase gastrointestinal stromal tumor cell invasion. Proc. Natl. Acad. Sci. USA.

[48] Purushothaman A., Bandari S.K., Liu J., Mobley J.A., Brown E.E., Sanderson R.D. (2016). Fibronectin on the Surface of Myeloma Cell-derived Exosomes Mediates Exosome-Cell Interactions. J. Biol. Chem..

[49] Lundy S.K., Klinker M.W., Fox D.A. (2015). Killer B lymphocytes and their fas ligand positive exosomes as inducers of immune tolerance. Front. Immunol..

[50] Yamashita T., Kamada H., Kanasaki S., Maeda Y., Nagano K., Abe Y., Inoue M., Yoshioka Y., Tsutsumi Y., Katayama S. (2013). Epidermal growth factor receptor localized to exosome membranes as a possible biomarker for lung cancer diagnosis. Pharmazie.

[51] Higginbotham J.N., Beckler M.D., Gephart J.D., Franklin J.L., Bogatcheva G., Kremers G.J., Piston D.W., Ayers G.D., McConnell R.E., Tyska M.J. (2011). Amphiregulin exosomes increase cancer cell invasion. Curr. Biol..

[52] Srinivasan S., Su M., Ravishankar S., Moore J., Head P., Dixon J.B., Vannberg F. (2017). TLR-exosomes exhibit distinct kinetics and effector function. Sci. Rep..

[53] Gross J.C., Chaudhary V., Bartscherer K., Boutros M. (2012). Active Wnt proteins are secreted on exosomes. Nat. Cell Biol..

[54] McBride J.D., Rodriguez-Menocal L., Guzman W., Candanedo A., Garcia-Contreras M., Badiavas E.V. (2017). Bone Marrow Mesenchymal Stem Cell-Derived CD63(+) Exosomes Transport Wnt3a Exteriorly and Enhance Dermal Fibroblast Proliferation, Migration, and Angiogenesis In Vitro. Stem Cells Dev..

[55] Pasquale E.B. (2016). Exosomes expand the sphere of influence of Eph receptors and ephrins. J. Cell Biol..

[56] Wada J., Onishi H., Suzuki H., Yamasaki A., Nagai S., Morisaki T., Katano M. (2010). Surface-bound TGF-beta-1 on effusion-derived exosomes participates in maintenance of number and suppressive function of regulatory T-cells in malignant effusions. Anticancer. Res..

[57] Shelke G.V., Yin Y., Jang S.C., Lässer C., Wennmalm S., Hoffmann H.J., Li L., Gho Y.S., Nilsson J.A., Lötvall J. (2019). Endosomal signaling via exosome surface TGFb. J. Extracell. Vesicles.

[58] Cossetti C., Iraci N., Mercer T.R., Leonardi T., Alpi E., Drago D., Alfaro-Cervello C., Saini H.K., Davis M.P., Schaeffer J. (2014). Extracellular vesicles from neural stem cells transfer IFN-γ via Ifngr1 to activate Stat1 signaling in target cells. Mol. Cell.

[59] Johnstone R.M., Bianchini A., Teng K. (1989). Reticulocyte maturation and exosome release: Transferrin receptor containing exosomes shows multiple plasma membrane functions. Blood.

[60] Nasca C., Dobbin J., Bigio B., Watson K., de Angelis P., Kautz M., Cochran A., Mathé A.A., Kocsis J.H., Lee F.S. (2020). Insulin receptor substrate in brain-enriched exosomes in subjects with major depression: On the path of creation of biosignatures of central insulin resistance. Mol. Psychiatry.

[61] Nazimek K., Bryniarski K., Ptak W., Groot Kormelink T., Askenase P.W. (2020). Orally administered, antigen-specific, T and B cell-derived suppressor exosomes deliver miRNA-150 to strongly inhibit DTH by binding to peptides in MHC of APC due to antibody light chain coating. Int. J. Mol. Sci..

[62] Nazimek K., Bustos-Morán E., Blas-Rus N., Nowak B., Totoń-Żurańska J., Seweryn M., Wołkow M.W.P., Askenase P.W., Sánchez-Madrid F., Bryniarski K. (2021). Regulation at the immune synapse by a multi-exosome-miRNA circuit of APC-connected T cells. Pharmaceuticals.

[63] Wąsik M., Nazimek K., Nowak B., Askenase P.W., Bryniarski K. (2019). Delayed-Type Hypersensitivity Underlying Casein Allergy Is Suppressed by Extracellular Vesicles Carrying miRNA-150. Nutrients.

[64] Bryniarski K., Ptak W., Jayakumar A., Püllmann K., Caplan M.J., Chairoungdua A., Lu J., Adams B.D., Sikora E., Nazimek K. (2013). Antigen-specific, antibody-coated, exosome-like nanovesicles deliver suppressor T-cell microRNA-150 to effector T cells to inhibit contact sensitivity. J. Allergy Clin. Immunol..

[65] Blanchard N., Lankar D., Faure F., Regnault A., Dumont C., Raposo G., Hivroz C. (2002). TCR Activation of Human T Cells Induces the Production of Exosomes Bearing the TCR/CD3/ζ Complex. J. Immunol..

[66] Choudhuri K., Llodrá J., Roth E.W., Tsai J., Gordo S., Wucherpfennig K.W., Kam L.C., Stokes D.L., Dustin M.L. (2014). Polarized release of T- cell-receptor-enriched microvesicles at the immunological synapse. Nature.

[67] Zarà M., Guidetti G.F., Camera M., Canobbio I., Amadio P., Torti M., Tremoli E., Barbieri S.S. (2019). Biology and Role of Extracellular Vesicles (EVs) in the Pathogenesis of Thrombosis. Int. J. Mol. Sci..

[68] Muhsin-Sharafaldine M.R., Saunderson S.C., Dunn A.C., Faed J.M., Kleffmann T., McLellan A.D. (2016). Procoagulant and immunogenic properties of melanoma exosomes, microvesicles and apoptotic vesicles. Oncotarget.

[69] Brzozowski J.S., Bond D.R., Jankowski H., Goldie B.J., Burchell R., Naudin C., Smith N.D., Scarlett C.J., Larsen M.R., Dun M.D. (2018). Extracellular vesicles with altered tetraspanin CD9 and CD151 levels confer increased prostate cell motility and invasion. Sci. Rep..

[70] Rana S., Zöller M. (2011). The Functional Importance of Tetraspanins in Exosomes. Biochem. Soc. Trans..

[71] Matsumoto A., Takahashi Y., Nishikawa M., Sano K., Morishita M., Charoenviriyakul C., Saji H., Takakura Y. (2017). Role of Phosphatidylserine-Derived Negative Surface Charges in the Recognition and Uptake of Intravenously Injected B16BL6-Derived Exosomes by Macrophages. J. Pharm. Sci..

[72] Balaj L., Atai N.A., Chen W., Mu D., Tannous B.A., Breakefield X.O., Skog J., Maguire C.A. (2015). Heparin affinity purification of extracellular vesicles. Sci. Rep..

[73] Popa S.J., Stewart S.E., Moreau K. (2018). Unconventional secretion of annexins and galectins. Semin. Cell Dev. Biol..

[74] Jeppesen D.K., Fenix A.M., Franklin J.L., Higginbotham J.N., Zhang Q., Zimmerman L.J., Liebler D.C., Ping J., Liu Q., Evans R. (2019). Reassessment of Exosome composition. Cell.

[75] Poggio M., Hu T., Pai C.C., Chu B., Belair C.D., Chang A., Montabana E., Lang U.E., Fu Q., Fong L. (2019). Suppression of Exosomal PD-L1 Induces Systemic Anti-tumor Immunity and Memory. Cell.

[76] Chen G., Huang A.C., Zhang W., Zhang G., Wu M., Xu W., Yu Z., Yang J., Wang B., Sun H. (2018). Exosomal PD-L1 contributes to immunosuppression and is associated with anti-PD-1 response. Nature.

[77] Milosevits G., Szebeni J., Krol S. (2015). Exosomes: Potential model for complement—Stealth delivery systems. Eur. J. Nanomed..

[78] Karasu E., Eisenhardt S.U., Harant J., Huber-Lang M. (2018). Extracellular Vesicles: Packages Sent With Complement. Front. Immunol..

[79] Cerezo-Magaña M., Bång-Rudenstam A., Belting M. (2019). The pleiotropic role of proteoglycans in extracellular vesicle mediated communication in the tumor microenvironment. Semin. Cancer Biol..

[80] Song X., Ding Y., Liu G., Yang X., Zhao R., Zhang Y., Zhao X., Anderson G.J., Nie G. (2016). Cancer Cell-derived Exosomes Induce Mitogen-activated Protein Kinase-dependent Monocyte Survival by Transport of Functional Receptor Tyrosine Kinases. J. Biol. Chem..

[81] Groth E., Pruessmeyer J., Babendreyer A., Schumacher J., Pasqualon T., Dreymueller D., Higashiyama S., Lorenzen I., Grötzinger J., Cataldo D. (2016). Stimulated release and functional activity of surface expressed metalloproteinase ADAM17 in exosomes. Biochim. Biophys. Acta.

[82] Shenoy G.N., Loyall J., Berenson C.S., Kelleher R.J., Iyer V., Balu-Iyer S.V., Odunsi K., Bankert R.B. (2018). Sialic Acid−Dependent Inhibition of T Cells by Exosomal Ganglioside GD3 in Ovarian Tumor Microenvironments. J. Immunol..

[83] Paolini L., Orizio F., Busatto S., Radeghieri A., Bresciani R., Bergese P., Monti E. (2017). Exosomes Secreted by HeLa Cells Shuttle on Their Surface the Plasma Membrane-Associated Sialidase NEU3. Biochemistry.

[84] Escrevente C., Grammel N., Kandzia S., Zeiser J., Tranfield E.M., Conradt H.S., Costa J. (2013). Sialoglycoproteins and N-Glycans from Secreted Exosomes of Ovarian Carcinoma Cells. PLoS ONE.

[85] Németh A., Orgovan N., Sódar B.W., Osteikoetxea X., Pálóczi K., Szabó-Taylor K.É., Vukman K.V., Kittel Á., Turiák L., Wiener Z. (2017). Antibiotic- induced release of small extracellular vesicles (exosomes) with surface-associated DNA. Sci. Rep..

[86] Wan S., Zhang L., Wang S., Liu Y., Wu C., Cui C., Sun H., Shi M., Jiang Y., Li L. (2017). Molecular Recognition-Based DNA Nanoassemblies on the Surfaces of Nanosized Exosomes. J. Am. Chem. Soc..

[87] Malkin E.Z., Bratman S.V. (2020). Bioactive DNA from extracellular vesicles and particles. Cell Death Dis..

[88] Wang W., Zhu N., Yan T., Shi Y.N., Chen J., Zhang C.J., Xie X.J., Liao D.F., Qin L. (2020). The crosstalk: Exosomes and lipid metabolism. Cell Commun. Signal..

[89] Hawari F.I., Rouhani F.N., Cui X., Yu Z.X., Buckley C., Kaler M., Levine S.J. (2004). Release of full-length 55-kDa TNF receptor 1 in exosome-like vesicles: A mechanism for generation of soluble cytokine receptors. PNAS.

[90] Zhang J., Hawari F.I., Shamburek R.D., Adamik B., Kaler M., Islam A., Liao D.W., Rouhani F.N., Ingham M., Levine S.J. (2008). Circulating TNFR1 exosome-like vesicles partition with the LDL fraction of human plasma. Biochem. Biophys. Res. Commun..

[91] Zhang H., Freitas D., Kim H.S., Fabijanic K., Li Z., Chen H., Mark M.T., Molina H., Martin A.B., Bojmar L. (2018). Identification of distinct nanoparticles and subsets of extracellular vesicles by asymmetric flow field-flow fractionation. Nat. Cell Biol..

[92] Mastoridis S., Bertolino G.M., Whitehouse G., Dazzi F., Sanchez-Fueyo A., Martinez-Llordella M. (2018). Multiparametric Analysis of Circulating Exosomes and Other Small Extracellular Vesicles by Advanced Imaging Flow Cytometry. Front. Immunol..

[93] Liu S.L., Sun P., Li Y., Liu S.S., Lu Y. (2019). Exosomes as critical mediators of cell-to-cell communication in cancer pathogenesis and their potential clinical application. Transl. Cancer Res..

[94] Batagov A.O., Kurochkin I.V. (2013). Exosomes secreted by human cells transport largely mRNA fragments that are enriched in the 3′-untranslated regions. Biol. Direct.

[95] Ghisi M., Corradin A., Basso K., Frasson C., Serafin V., Mukherjee S., Mussolin L., Ruggero K., Bonanno L., Guffanti A. (2011). Modulation of microRNA expression in human T-cell development: Targeting of NOTCH3 by miR-150. Blood.

[96] Neilson J.R., Zheng G.X., Burge C.B., Sharp P.A. (2007). Dynamic regulation of miRNA expression in ordered stages of cellular development. Genes Dev..

[97] Pálfy M., Reményi A., Korcsmáros T. (2012). Endosomal crosstalk: Meeting points for signaling pathways. Trends Cell Biol..

[98] Ung T.H., Madsen H.J., Hellwinkel J.E., Lencioni A.M., Graner M.W. (2014). Exosome proteomics reveals transcriptional regulator proteins with potential to mediate downstream pathways. Cancer Sci..

[99] Lankford K.L., Arroyo E.J., Nazimek K., Bryniarski K., Askenase P.W., Kocsis J.D. (2018). Intravenously Delivered Mesenchymal Stem Cell-Derived Exosomes Specifically Target M2-type Macrophages of the Injured Spinal Cord. PLoS ONE.

[100] Cvjetkovic A., Jang S.C., Konečná B., Höög J.L., Sihlbom C., Lässer C., Lötvall J. (2016). Detailed Analysis of Protein Topology of Extracellular Vesicles– Evidence of Unconventional Membrane Protein Orientation. Sci. Rep..

[101] Heusermann W., Hean J., Trojer D., Steib E., Von Bueren S., Graff-Meyer A., Genoud C., Martin K., Pizzato N., Voshol J. (2016). Exosomes surf on filopodia to enter cells at endocytic hot spots, traffic within endosomes, and are targeted to the ER. J. Cell Biol..

[102] Feng D., Zhao W.L., Ye Y.Y., Bai X.C., Liu R.Q., Chang L.F., Zhou Q., Sui S.F. (2010). Cellular internalization of exosomes occurs through phagocytosis. Traffic.

[103] Tian T., Zhu Y.L., Zhou Y.Y., Liang G.F., Wang Y.Y., Hu F.H., Xiao Z.D. (2014). Exosome uptake through clathrin-mediated endocytosis and macropinocytosis and mediating miR-21 delivery. J. Biol. Chem..

[104] Fitzner D., Schnaars M., Van Rossum D., Krishnamoorthy G., Dibaj P., Bakhti M., Regen T., Hanisch U.K., Simons M. (2011). Selective transfer of exosomes from oligodendrocytes to microglia by macropinocytosis. J. Cell Sci..

[105] Svensson K.J., Christianson H.C., Wittrup A., Bourseau-Guilmain E., Lindqvist E., Svensson L.M., Mörgelin M., Belting M. (2013). Exosome uptake depends on ERK1/2-heat shock protein 27 signaling and lipid Raft-mediated endocytosis negatively regulated by caveolin-1. J. Biol. Chem..

[106] Roberts-Dalton H.D., Cocks A., Falcon-Perez J.M., Sayers E.J., Webber J.P., Watson P., Clayton A., Jones A.T. (2017). Fluorescence labelling of extracellular vesicles using a novel thiol-based strategy for quantitative analysis of cellular delivery and intracellular traffic. Nanoscale.

[107] Izumi H., Kosaka N., Shimizu T., Sekine K., Ochiya T., Takase M. (2012). Bovine milk contains microRNA and messenger RNA that are stable under degradative conditions. J. Dairy Sci..

[108] Pieters B.C., Arntz O.J., Bennink M.B., Broeren M.G., van Caam A.P., Koenders M.I., van Lent P.L., van den Berg W.B., de Vries M., van der Kraan P.M. (2015). Commercial cow milk contains physically stable extracellular vesicles expressing immunoregulatory TGF-β. PLoS ONE.

[109] Luo Y., Wang P., Wang X., Wang Y., Mu Z., Li Q., Fu Y., Xiao J., Li G., Ma Y. (2017). Detection of dietetically absorbed maize-derived microRNAs in pigs. Sci. Rep..

[110] Gu Y., Li M., Wang T., Liang Y., Zhong Z., Wang X., Zhou Q., Chen L., Lang Q., He Z. (2012). Lactation- related microRNA expression profiles of porcine breast milk exosomes. PLoS ONE.

[111] Melnik B.C., John S.M., Schmitz G. (2014). Milk: An exosomal microRNA transmitter promoting thymic regulatory T cell maturation preventing the development of atopy?. J. Transl. Med..

[112] Benmoussa A., Lee C.H.C., Laffont B., Savard P., Laugier J., Boilard E., Gilbert C., Fliss I., Provost P. (2016). Commercial dairy cow milk microRNAs resist digestion under simulated gastrointestinal tract conditions. J. Nutr..

[113] Admyre C., Johansson S.M., Qazi K.R., Filén J.J., Lahesmaa R., Norman M., Neve E.P., Scheynius A., Gabrielsson S. (2007). Exosomes with immune modulatory features are present in human breast milk. J. Immunol..

[114] Zhou Q., Li M., Wang X., Li Q., Wang T., Zhu Q., Zhou X., Wang X., Gao X., Li X. (2012). Immune-related microRNAs are abundant in breast milk exosomes. Int. J. Biol. Sci..

[115] Denzler R., Stoffel M. (2015). Uptake and function studies of maternal milk-derived microRNAs. J. Biol. Chem..

[116] Melnik B.C., John S.M., Schmitz G. (2013). Milk is not just food but most likely a genetic transfection system activating mTORC1 signaling for postnatal growth. Nutr. J..

[117] Durak-Kozica M., Baster Z., Kubat K., Stępień E. (2018). 3D visualization of extracellular vesicle uptake by endothelial cells. Cell. Mol. Biol. Lett..

[118] Plebanek M.P., Mutharasan R.K., Volpert O., Matov A., Gatlin J.C., Thaxton C.S. (2015). Nanoparticle targeting and cholesterol flux through scavenger receptor type B-1 inhibits cellular exosome uptake. Sci. Rep..

[119] Zomer A., Maynard C., Verweij F.J., Kamermans A., Schäfer R., Beerling E., Schiffelers R.M., De Wit E., Berenguer J., Ellenbroek S.I.J. (2015). In vivo imaging reveals extracellular vesicle-mediated phenocopying of metastatic behavior. Cell.

[120] Lai C.P., Kim E.Y., Badr C.E., Weissleder R., Mempel T.R., Tannous B.A., Breakefield X.O. (2015). Visualization and tracking of tumour extracellular vesicle delivery and RNA translation using multiplexed reporters. Nat. Commun..

[121] Van Dongen H.M., Masoumi N., Witwer K.W., Pegtel D.M. (2016). Extracellular vesicles exploit viral entry routes for cargo delivery. Microbiol. Mol. Biol. Rev..

[122] Alvarez-Erviti L., Seow Y., Schapira A.H., Gardiner C., Sargent I.L., Wood M.J., Cooper J.M. (2011). Lysosomal dysfunction increases exosome-mediated alpha-synuclein release and transmission. Neurobiol. Dis..

[123] Mittelbrunn M., Gutiérrez-Vázquez C., Villarroya-Beltri C., González S., Sánchez-Cabo F., González M.Á., Bernad A., Sánchez-Madrid F. (2011). Unidirectional transfer of microRNA-loaded exosomes from T cells to antigen-presenting cells. Nat. Commun..

[124] Momen-Heravi F., Bala S., Kodys K., Szabo G. (2015). Exosomes derived from alcohol-treated hepatocytes horizontally transfer liver specific miRNA-122 and sensitize monocytes to LPS. Sci. Rep..

[125] Roccaro A.M., Sacco A., Maiso P., Azab A.K., Tai Y.T., Reagan M., Azab F., Flores L.M., Campigotto F., Weller E. (2013). BM mesenchymal stromal cell–derived exosomes facilitate multiple myeloma progression. J. Clin. Investig..

[126] Suetsugu A., Honma K., Saji S., Moriwaki H., Ochiya T., Hoffman R.M. (2013). Imaging exosome transfer from breast cancer cells to stroma at metastatic sites in orthotopic nude-mouse models. Adv. Drug Deliv. Rev..

[127] Liu Y., Li D., Liu Z., Zhou Y., Chu D., Li X., Jiang X., Hou D., Chen X., Chen Y. (2015). Targeted exosome- mediated delivery of opioid receptor Mu siRNA for the treatment of morphine relapse. Sci. Rep..

[128] Tian T., Zhu Y.L., Hu F.H., Wang Y.Y., Huang N.P., Xiao Z.D. (2013). Dynamics of exosome internalization and trafficking. J. Cell. Physiol..

[129] Attie A.D., Raines R.T. (1995). Analysis of Receptor-Ligand Interactions. J. Chem. Educ..

[130] Loeb J.N., Strickland S. (1987). Hormone binding and coupled response relationships in systems dependent on the generation of secondary mediators. Mol. Endocrinol..

[131] Lomash S., Nagpal S., Salunke D.M. (2010). An antibody as surrogate receptor reveals determinants of activity of an innate immune peptide antibiotic. J. Biol. Chem..

[132] Godfrey-Smith P. (2013). Philosophy of Biology.

[133] Jones R.H. (2000). Reductionism: Analysis and the Fullness of Reality.

[134] Van Regenmortel M.H. (2004). Biological complexity emerges from the ashes of genetic reductionism. J. Mol. Recognit..

[135] King M.R. (2016). Commentary: Basic Research in HIV Vaccinology Is Hampered by Reductionist Thinking. Front. Immunol..

[136] Ahn A.C., Tewari M., Poon C.S., Phillips R.S. (2006). The limits of reductionism in medicine: Could systems biology offer an alternative?. PLoS Med..

[137] Kesić S. (2016). Systems biology, emergence and antireductionism. Saudi J. Biol. Sci..

[138] Mesarovic M.D., Sreenath S.N., Keene J.D. (2004). Search for organising principles: Understanding in systems biology. Syst. Biol..

[139] Van Vranken J.G., Rutter J. (2016). The whole (cell) is less than the sum of its parts. Cell.

[140] Kitano H. (2002). Systems biology: A brief overview. Science.

[141] Wanjek C. (2011). Systems biology as defined by NIH: An intellectual resource for integrative biology. NIH Catal..

[142] Ayers D., Day P.J. (2015). Systems medicine: The application of systems biology approaches for modern medical research and drug development. Medical Research and Drug Development. Mol. Biol. Int..

[143] Sharma A. (2015). Systems genomics analysis centered on epigenetic inheritance supports development of a unified theory of biology. J. Exp. Biol..

[144] Muller L., Simms P., Hong C.S., Nishimura M.I., Jackson E.K., Watkins S.C., Whiteside T.L. (2017). Human tumor-derived exosomes (TEX) regulate Treg functions via cell surface signaling rather than uptake mechanisms. Oncoimmunology.

[145] Ganser L.R., Kelly M.L., Herschlag D., Al-Hashimi H.M. (2019). The roles of structural dynamics in the cellular functions of RNAs. Nat. Rev. Mol. Cell Biol..

[146] Bergthaler A., Menche J. (2017). The immune system as a social network. Nat. Immunol..

[147] Morishita M., Takahashi Y., Nishikawa M., Takakura Y. (2017). Pharmacokinetics of Exosomes-An Important Factor for Elucidating the Biological Roles of Exosomes and for the Development of Exosome-Based Therapeutics. J. Pharm. Sci..

[148] Charoenviriyakul C., Takahashi Y., Morishita M., Nishikawa M., Takakura Y. (2018). Role of Extracellular Vesicle Surface Proteins in the Pharmacokinetics of Extracellular Vesicles. Mol. Pharm..

[149] Cataldi M., Vigliotti C., Mosca T., Cammarota M., Capone D. (2017). Emerging Role of the Spleen in the Pharmacokinetics of Monoclonal Antibodies, Nanoparticles and Exosomes. Int. J. Mol. Sci..

[150] Agarwal U., George A., Bhutani S., Ghosh-Choudhary S., Maxwell J.T., Brown M.E., Mehta Y., Platt M.O., Liang Y., Sahoo S. (2017). Experimental, Systems, and Computational Approaches to Understanding the MicroRNA-Mediated Reparative Potential of Cardiac Progenitor Cell- Derived Exosomes From Pediatric Patients. Circ. Res..

[151] Gézsi A., Kovács Á., Visnovitz T., Buzás E.I. (2019). Systems biology approaches to investigating the roles of extracellular vesicles in human diseases. Exp. Mol. Med..

[152] Conlan R.S., Pisano S., Oliveira M.I., Ferrari M., Pinto I.M. (2017). Exosomes as Reconfigurable Therapeutic Systems. Trends Mol. Med..

[153] Deem M.W. (2004). Complexity in the immune system: New opportunities for chemical engineering research. AIChE J..

[154] Hoek K., Howard L., Allos T., Samir P., Niu X., Creech B., Edwards K., Link A. (2013). Systems biology assessment of human immune responses after seasonal trivalent inactivated influenza vaccine (P4307). J. Immunol..

[155] Subramanian N., Torabi-Parizi P., Gottschalk R.A., Germain R.N., Dutta B. (2015). Network representations of immune system complexity. Wiley Interdiscip. Rev. Syst. Biol. Med..

[156] Dudek N.L., Croft N.P., Schittenhelm R.B., Ramarathinam S.H., Purcell A.W. (2016). A Systems Approach to Understand Antigen Presentation and the Immune Response. Methods Mol. Biol..

[157] Davis M.M., Tato C.M., Furman D. (2017). Systems immunology: Just getting started. Nat. Immunol..

[158] Germain R.N. (2018). How Can Systems Biology Test Principles and Tools Using Immune Cells as a Model?. Cell Syst..

[159] Germain R.N., Greenbaum B.D., Hoffmann A., Miller-Jensen K., Mora T., Walczak A.M., Segal E., Vogl T., Klompus S., Peled-Liviatan S. (2018). What Can Immunologists Learn from Systems Approaches?. Trends Immunol..

[160] Kalra H., Adda C.G., Liem M., Ang C.S., Mechler A., Simpson R.J., Hulett M.D., Mathivanan S. (2013). Comparative proteomics evaluation of plasma exosome isolation techniques and assessment of the stability of exosomes in normal human blood plasma. Proteomics.

[161] Sharma A. (2014). Bioinformatic analysis revealing association of exosomal mRNAs and proteins in epigenetic inheritance. J. Theor. Biol..

[162] Pathan M., Keerthikumar S., Chisanga D., Alessandro R., Ang C.S., Askenase P., Batagov A.O., Benito-Martin A., Camussi G., Clayton A. (2017). A novel community driven software for functional enrichment analysis of extracellular vesicles data. J. Extracell. Vesicles.

[163] Van Deun J., Mestdagh P., Agostinis P., Akay Ö., Anand S., Anckaert J., Martinez Z.A., Baetens T., Beghein E., Bertier L. (2017). EV-TRACK: Transparent reporting and centralizing knowledge in extracellular vesicle research. Nat. Methods.

[164] Cheung K.H., Keerthikumar S., Roncaglia P., Subramanian S.L., Roth M.E., Samuel M., Anand S., Gangoda L., Gould S., Alexander R. (2016). Extending gene ontology in the context of extracellular RNA and vesicle communication. J. Biomed. Semant..

[165] Bobrie A., Théry C. (2013). Unraveling the physiological functions of exosome secretion by tumors. Oncoimmunology.

[166] Budnik V., Ruiz-Cañada C., Wendler F. (2016). Extracellular vesicles round off communication in the nervous system. Nat. Rev. Neurosci..

[167] Hill A.F., Pegtel D.M., Lambertz U., Leonardi T., O’driscoll L., Pluchino S., Ter-Ovanesyan D., Nolte-‘t Hoen E.N. (2013). ISEV position paper: Extracellular vesicle RNA analysis and bioinformatics. J. Extracell. Vesicles.

[168] Nayfach S., Pollard K.S. (2016). Toward accurate and quantitative comparative metagenomics. Cell.

[169] Shao H., Chung J., Balaj L., Charest A., Bigner D.D., Carter B.S., Hochberg F.H., Breakefield X.O., Weissleder R., Lee H. (2012). Protein typing of circulating microvesicles allows real-time monitoring of glioblastoma therapy. Nat. Med..

[170] Zhang J., Li S., Li L., Li M., Guo C., Yao J., Mi S. (2015). Exosome and exosomal microRNA: Trafficking, sorting, and function. Genom. Proteom. Bioinform..

[171] Pisitkun T., Gandolfo M.T., Das S., Knepper M.A., Bagnasco S.M. (2012). Application of systems biology principles to protein biomarker discovery: Urinary exosomal proteome in renal transplantation. Proteom. -Clin. Appl..

[172] Pathan M., Keerthikumar S., Ang C.S., Gangoda L., Quek C.Y., Williamson N.A., Mouradov D., Sieber O.M., Simpson R.J., Salim A. (2015). FunRich: An open access standalone functional enrichment and interaction network analysis tool. Proteomics.

[173] Chen Y., Xie Y., Xu L., Zhan S., Xiao Y., Gao Y., Wu B., Ge W. (2017). Protein content and functional characteristics of serum-purified exosomes from patients with colorectal cancer revealed by quantitative proteomics. Int. J. Cancer.

[174] Kirschbaum K., Sonner J.K., Zeller M.W., Deumelandt K., Bode J., Sharma R., Krüwel T., Fischer M., Hoffmann A., Da Silva M.C. (2016). In vivo nanoparticle imaging of innate immune cells can serve as a marker of disease severity in a model of multiple sclerosis. Proc. Natl. Acad. Sci. USA.

[175] Koh Y.Q., Peiris H.N., Vaswani K., Reed S., Rice G.E., Salomon C., Mitchell M.D. (2016). Characterization of exosomal release in bovine endometrial intercaruncular stromal cells. Reprod. Biol. Endocrinol..

[176] Chisanga D., Keerthikumar S., Pathan M., Ariyaratne D., Kalra H., Boukouris S., Mathew N.A., Saffar H.A., Gangoda L., Ang C.S. (2015). Colorectal cancer atlas: An integrative resource for genomic and proteomic annotations from colorectal cancer cell lines and tissues. Nucleic Acids Res..

[177] Lai X., Wolkenhauer O., Vera J. (2016). Understanding microRNA-mediated gene regulatory networks through mathematical modelling. Nucleic Acids Res..

[178] Sadovska L., Bajo Santos C., Kalniņa Z., Line A. (2015). Biodistribution, uptake and effects caused by cancer-derived extracellular vesicles. J. Circ. Biomark..

[179] Zaborowski M.P., Balaj L., Breakefield X.O., Lai C.P. (2015). Extracellular vesicles: Composition, biological relevance, and methods of study. Bioscience.

[180] Johnson G.D., Mackie P., Jodar M., Moskovtsev S., Krawetz S.A. (2015). Chromatin and extracellular vesicle associated sperm RNAs. Nucleic Acids Res..

[181] Lin Y., Wu J., Gu W., Huang Y., Tong Z., Huang L., Tan J. (2018). Exosome-Liposome Hybrid Nanoparticles Deliver CRISPR/Cas9 System in MSCs. Adv. Sci..

[182] Ono R., Yasuhiko Y., Aisaki K.I., Kitajima S., Kanno J., Hirabayashi Y. (2019). Exosome-mediated horizontal gene transfer occurs in double-strand break repair during genome editing. Commun. Biol..

[183] Ye Y., Zhang X., Xie F., Xu B., Xie P., Yang T., Shi Q., Zhang C.Y., Zhang Y., Chen J. (2020). An engineered exosome for delivering sgRNA:Cas9 ribonucleoprotein complex and genome editing in recipient cells. Biomater. Sci..

[184] Zhang L., Hou D., Chen X., Li D., Zhu L., Zhang Y., Li J., Bian Z., Liang X., Cai X. (2012). Exogenous plant MIR168a specifically targets mammalian LDLRAP1: Evidence of cross-kingdom regulation by microRNA. Cell Res..

[185] Lian Q., Xu J., Yan S., Huang M., Ding H., Sun X., Bi A., Ding J., Sun B., Geng M. (2017). Chemotherapy-induced intestinal inflammatory responses are mediated by exosome secretion of double-strand DNA via AIM2 inflammasome activation. Cell Res..

[186] Beatty M., Guduric-Fuchs J., Brown E., Bridgett S., Chakravarthy U., Hogg R.E., Simpson D.A. (2014). Small RNAs from plants, bacteria and fungi within the order Hypocreales are ubiquitous in human plasma. BMC Genom..

[187] Mu J., Zhuang X., Wang Q., Jiang H., Deng Z.B., Wang B., Zhang L., Kakar S., Jun Y., Miller D. (2014). Interspecies communication between plant and mouse gut host cells through edible plant derived exosome-like nanoparticles. Mol. Nutr. Food Res..

[188] Pastrello C., Tsay M., McQuaid R., Abovsky M., Pasini E., Shirdel E., Angeli M., Tokar T., Jamnik J., Kotlyar M. (2017). Circulating plant miRNAs can regulate human gene expression in vitro. Sci. Rep..

[189] Chin A.R., Fong M.Y., Somlo G., Wu J., Swiderski P., Wu X., Wang S.E. (2016). Cross-kingdom inhibition of breast cancer growth by plant miR159. Cell Res..

[190] Dickinson B., Zhang Y., Petrick J.S., Heck G., Ivashuta S., Marshall W.S. (2013). Lack of detectable oral bioavailability of plant microRNAs after feeding in mice. Nat. Biotechnol..

[191] Snow J.W., Hale A.E., Isaacs S.K., Baggish A.L., Chan S.Y. (2013). Ineffective delivery of diet- derived microRNAs to recipient animal organisms. RNA Biol..

[192] Witwer K.W., McAlexander M.A., Queen S.E., Adams R.J. (2013). Real-time quantitative PCR and droplet digital PCR for plant miRNAs in mammalian blood provide little evidence for general uptake of dietary miRNAs: Limited evidence for general uptake of dietary plant xenomiRs. RNA Biol..

[193] Witwer K. (2018). Alternative miRNAs? Human sequences misidentified as plant miRNAs in plant studies and in human plasma. F1000Research.

[194] Micó V., Martín R., Lasunción M.A., Ordovás J.M., Daimiel L. (2016). Unsuccessful detection of plant microRNAs in beer, extra virgin olive oil and human plasma after an acute ingestion of extra virgin olive oil. Plant Foods Hum. Nutr..

[195] Mar-Aguilar F., Arreola-Triana A., Mata-Cardona D., Gonzalez-Villasana V., Rodríguez-Padilla C., Reséndez-Pérez D. (2020). Evidence of transfer of miRNAs from the diet to the blood still inconclusive. PeerJ.

[196] Kang W., Bang-Berthelsen C.H., Holm A., Houben A.J., Müller A.H., Thymann T., Pociot F., Estivill X., Friedländer M.R. (2017). Survey of 800+ data sets from human tissue and body fluid reveals xenomiRs are likely artifacts. RNA.

[197] Liang H., Zhang S., Fu Z., Wang Y., Wang N., Liu Y., Zhao C., Wu J., Hu Y., Zhang J. (2015). Effective detection and quantification of dietetically absorbed plant microRNAs in human plasma. J. Nutr. Biochem..

[198] Zhang S., Sang X., Hou D., Chen J., Gu H., Zhang Y., Li J., Yang D., Zhu H., Yang X. (2019). Plant-derived RNAi therapeutics: A strategic inhibitor of HBsAg. Biomaterials.

[199] Liu Y.C., Chen W.L., Kung W.H., Huang H.D. (2017). Plant miRNAs found in human circulating system provide evidences of cross kingdom RNAi. BMC Genom..

[200] Zhou G., Zhou Y., Chen X. (2017). New Insight into Inter-kingdom Communication: Horizontal Transfer of Mobile Small RNAs. Front. Microbiol..

[201] Hirschi K.D. (2017). Navigating dietary small RNAs. Genes Nutr..

[202] Perge P., Nagy Z., Decmann Á., Igaz I., Igaz P. (2017). Potential relevance of microRNAs in inter-species epigenetic communication, and implications for disease pathogenesis. RNA Biol..

[203] Chan S.Y., Snow J.W. (2017). Uptake and impact of natural diet-derived small RNA in invertebrates: Implications for ecology and agriculture. RNA Biol..

[204] Sharma A., Sahu S., Kumari P., Gopi S.R., Malhotra R., Biswas S. (2017). Genome- wide identification and functional annotation of miRNAs in anti-inflammatory plant and their cross-kingdom regulation in Homo sapiens. J. Biomol. Struct. Dyn..

[205] Zhang H., Li Y., Liu Y., Liu H., Wang H., Jin W., Zhang Y., Zhang C., Xu D. (2016). Role of plant MicroRNA in cross-species regulatory networks of humans. BMC Syst. Biol..

[206] Philip A., Ferro V.A., Tate R.J. (2015). Determination of the potential bioavailability of plant microRNAs using a simulated human digestion process. Mol. Nutr. Food Res..

[207] Askenase P.W., Van Loveren H., Kraeuter-Kops S., Ron Y., Meade R., Theoharides T.C., Nordlund J.J., Scovern H., Gerhson M.D., Ptak W. (1983). Defective elicitation of delayed-type hypersensitivity in W/Wv and S1/S1d mast cell deficient mice. J. Immunol..

[208] Galli S.J., Hammel I. (1984). Unequivocal delayed hypersensitivity in mast cell-deficient and beige mice. Science.

[209] Soares R.P., Xander P., Costa A.O., Marcilla A., Menezes-Neto A., Del Portillo H., Witwer K., Wauben M., Nolte-‘t Hoen E., Olivier M. (2017). Highlights of the São PauloISEV workshop on extracellular vesicles in cross-kingdom communication. J. Extracell. Vesicles.

[210] Witwer K.W., Zhang C.Y. (2017). Diet-derived microRNAs: Unicorn or silver bullet? Studying the relationship between genetics and nutrition in the improvement of human health. Genes Nutr..

[211] Witwer K.W., Hirschi K.D. (2014). Transfer and functional consequences of dietary microRNAs in vertebrates: Concepts in search of corroboration: Negative results challenge the hypothesis that dietary xenomiRs cross the gut and regulate genes in ingesting vertebrates, but important questions persist. BioEssays News Rev. Mol. Cell. Dev. Biol..

[212] Huang H., Davis C.D., Wang T.T. (2018). Extensive degradation and low bioavailability of orally consumed corn miRNAs in mice. Nutrients.

[213] Lim L.P., Lau N.C., Garrett-Engele P., Grimson A., Schelter J.M., Castle J., Bartel D.P., Linsley P.S., Johnson J.M. (2005). Microarray analysis shows that some microRNAs downregulate large numbers of target mRNAs. Nature.

[214] Fritz J.V., Heintz-Buschart A., Ghosal A., Wampach L., Etheridge A., Galas D., Wilmes P. (2016). Sources and functions of extracellular small RNAs in human circulation. Annu. Rev. Nutr..

[215] Alfonsi R., Grassi L., Signore M., Bonci D. (2018). The Double Face of Exosome-Carried MicroRNAs in Cancer Immunomodulation. Int. J. Mol. Sci..

[216] Martin H.C., Wani S., Steptoe A.L., Krishnan K., Nones K., Nourbakhsh E., Vlassov A., Grimmond S.M., Cloonan N. (2014). Imperfect centered miRNA binding sites are common and can mediate repression of target mRNAs. Genome Biol..

[217] Lu L.F., Gasteiger G., Yu I.S., Chaudhry A., Hsin J.P., Lu Y., Bos P.D., Lin L.L., Zawislak C.L., Cho S. (2015). A Single miRNA-mRNA Interaction Affects the Immune Response in a Context- and Cell-Type-Specific Manner. Immunity.

[218] Oh M., Rhee S., Moon J.H., Chae H., Lee S., Kang J., Kim S. (2017). Literature-based condition- specific miRNA-mRNA target prediction. PLoS ONE.

[219] Bartel D.P. (2009). MicroRNAs: Target recognition and regulatory functions. Cell.

[220] Linsley P.S., Schelter J., Burchard J., Kibukawa M., Martin M.M., Bartz S.R., Johnson J.M., Cummins J.M., Raymond C.K., Dai H. (2007). Transcripts targeted by the microRNA-16 family cooperatively regulate cell cycle progression. Mol. Cell. Biol..

[221] Korla K., Arrigo P., Mitra C.K. (2013). Promoters, toll like receptors and microRNAs: A strange association. Indian J. Biochem. Biophys..

[222] Cipolla G.A. (2014). A non-canonical landscape of the microRNA system. Front. Genet..

[223] Bang C., Batkai S., Dangwal S., Gupta S.K., Foinquinos A., Holzmann A., Just A., Remke J., Zimmer K., Zeug A. (2014). Cardiac fibroblast-derived microRNA passenger strand-enriched exosomes mediate cardiomyocyte hypertrophy. J. Clin. Investig..

[224] Brümmer A., Hausser J. (2014). MicroRNA binding sites in the coding region of mRNAs: Extending the repertoire of post-transcriptional gene regulation. Bioessays.

[225] Wuchty S., Fontana W., Hofacker I.L., Schuster P. (1999). Complete suboptimal folding of RNA and the stability of secondary structures. Biopolymers.

[226] Janas T., Janas M.M., Sapoń K., Janas T. (2015). Mechanisms of RNA loading into exosomes. FEBS Lett..

[227] Leamy K.A., Yennawar N.H., Bevilacqua P.C. (2017). Cooperative RNA Folding under Cellular Conditions Arises From Both Tertiary Structure Stabilization and Secondary Structure Destabilization. Biochemistry.

[228] Liu B., Childs-Disney J.L., Znosko B.M., Wang D., Fallahi M., Gallo S.M., Disney M.D. (2016). Analysis of secondary structural elements in human microRNA hairpin precursors. BMC Bioinform..

[229] Nam S., Ryu H., Son W.J., Kim Y.H., Kim K.T., Balch C., Nephew K.P., Lee J. (2014). Mg2+ effect on argonaute and RNA duplex by molecular dynamics and bioinformatics implications. PLoS ONE.

[230] Dallaire P., Tan H., Szulwach K., Ma C., Jin P., Major F. (2016). Structural dynamics control the MicroRNA maturation pathway. Nucleic Acids Res..

[231] Eliscovich C., Shenoy S.M., Singer R.H. (2017). Imaging mRNA and protein interactions within neurons. Proc. Natl. Acad. Sci. USA.

[232] O’Brien J., Hayder H., Zayed Y., Peng C. (2018). Overview of MicroRNA Biogenesis, Mechanisms of Actions, and Circulation. Front. Endocrinol..

[233] Aslan C., Kiaie S.H., Zolbanin N.M., Lotfinejad P., Ramezani R., Kashanchi F., Jafari R. (2021). Exosomes for mRNA delivery: A novel biotherapeutic strategy with hurdles and hope. BMC Biotechnol..

[234] Seo J., Jin D., Choi C.H., Lee H. (2017). Integration of MicroRNA, mRNA, and Protein Expression Data for the Identification of Cancer-Related MicroRNAs. PLoS ONE.

[235] Tian X.J., Zhang H., Zhang J., Xing J. (2016). Reciprocal regulation between mRNA and microRNA enables a bistable switch that directs cell fate decisions. FEBS Lett..

[236] Schmitz U., Lai X., Winter F., Wolkenhauer O., Vera J., Gupta S.K. (2014). Cooperative gene regulation by microRNA pairs and their identification using a computational workflow. Nucleic Acids Res..

[237] Hu Z., Yu D., Almeida-Suhett C., Tu K., Marini A.M., Eiden L., Braga M.F., Zhu J., Li Z. (2012). Expression of miRNAs and their cooperative regulation of the pathophysiology in traumatic brain injury. PLoS ONE.

[238] Kehl T., Backes C., Kern F., Fehlmann T., Ludwig N., Meese E., Lenhof H.P., Keller A. (2017). About miRNAs, miRNA seeds, target genes and target pathways. Oncotarget.

[239] Kim D., Chang H.R., Baek D. (2017). Rules for functional microRNA targeting. BMB Rep..

